# Spermidine biosynthesis by hypervirulent *Francisella tularensis* promotes fitness and salvages adenine

**DOI:** 10.1128/jb.00616-25

**Published:** 2026-04-22

**Authors:** Yinshi Yue, Dhananjay Shinde, Robert Moore, Yangsheng Yu, Vinai Chittezham Thomas, Tomáš Helikar, Marilynn A. Larson

**Affiliations:** 1 Department of Pathology, Microbiology, and Immunology, University of Nebraska Medical Center, Omaha, Nebraska, USA; 2 Department of Biochemistry, University of Nebraska-Lincoln, Lincoln, Nebraska, USA; Southern University of Science and Technology, Shenzhen, Guangdong, China

**Keywords:** hypervirulent *Francisella tularensis*, tularemia, arginine and methionine metabolism, polyamine biosynthesis and acquisition, spermidine, spermine, putrescine, methylthioadenosine, adenine salvage

## Abstract

*Francisella tularensis* is a highly infectious Tier 1 select agent that causes tularemia, a potentially fatal disease. *F. tularensis* subspecies *tularensis* (type A), including subtypes A.I and A.II, and subspecies *holarctica* (type B) differ considerably in pathogenicity, with A.I strains being recognized as some of the most virulent bacterial pathogens known. We previously revealed that only the A.I clade can synthesize spermidine *de novo*. In this study, we show that deletion of spermidine synthase *speE* (Δ*speE*) in prototype A.I strain SCHU S4 reduced spermidine levels relative to wild-type SCHU S4, type B live vaccine strain (LVS), and LVS Δ*speE*. In conventional spermidine-containing chemically defined medium (cCDM), SCHU S4 and SCHU S4 Δ*speE* grew substantially faster than LVS and LVS Δ*speE*; however, SCHU S4 Δ*speE* grew slower than SCHU S4. In modified cCDM without spermidine, SCHU S4 Δ*speE* grew in a biphasic manner, which was alleviated by methylthioadenosine, adenine, or adenosine supplementation. Conversely, spermidine addition caused diauxic growth by LVS and LVS Δ*speE* and was inhibitory above 0.1 mM, while SCHU S4 replication was substantially and similarly promoted by spermidine or spermine supplementation, with 0.02 mM spermidine being sufficient for maximum growth enhancement. These previously unknown traits demonstrate that *de novo* spermidine synthesis by A.I strains salvages adenine and promotes fitness, while exogenous spermidine and spermine further increase the rapid replication rate of hypervirulent A.I SCHU S4. These *F. tularensis* A.I attributes are likely an adaptation to an intracellular environment that contributes to persistence, while outcompeting an effective immune response during an infection.

**IMPORTANCE:**

*Francisella tularensis* is one of the most pathogenic bacteria known due to a low infectious dose, expansive host range, and high rate of mortality. This zoonotic pathogen poses a serious risk to public health and, therefore, is considered a potential bioterrorism agent. Our study revealed that hypervirulent strains of *F. tularensis* endogenously produce spermidine, which promotes fitness and salvages adenine, while exogenous spermidine or spermine further enhances the rapid replication rate of this intracellular pathogen. A better understanding of the inherent traits that allow *F. tularensis* to persist and outcompete an effective immune response is needed and may provide insight into preventing lethal infections by other intracellular pathogens.

## INTRODUCTION

*Francisella tularensis* is a gram-negative bacterium that is classified as a Tier 1 category A select agent (1). This zoonotic and facultative intracellular pathogen has an infectious dose as low as 1 to 10 organisms and causes tularemia, which can be fatal if untreated (2). Transmission of tularemia can occur via various routes from infected arthropods or animals, as well as contaminated air, water, food, or soil (3). Substantial differences in pathogenicity exist between the *F. tularensis* subpopulations due in part to chromosomal rearrangements that have affected gene content and expression (4, 5). The two most clinically relevant *F. tularensis* clades include subspecies *tularensis* (also known as type A) and *holarctica* (also known as type B). Type A strains are further divided into subtypes A.I and A.II due to additional genomic and phenotypic differences, with the A.I strains being recognized as some of the most pathogenic bacteria known (6). The most widely studied *F. tularensis* strain is type B live vaccine strain (LVS) since this attenuated strain is pathogenic to animals but not humans and therefore is exempt from regulatory compliance. The use of attenuated LVS as a vaccine can provide some level of protection against a respiratory infection by hypervirulent *F. tularensis* A.I strains (i.e., prototype A.I strain SCHU S4) in humans (7). However, a more protective and reliable vaccine is needed that eliminates the possibility of reversion back to wild type, since the mechanism of LVS attenuation remains unknown.

In the development of a chemically defined medium (CDM) that would support growth of the fastidious pathogen *F. tularensis*, 13 amino acids, the polyamine spermidine or spermine, and other components were needed (8, 9). This CDM formulation was further optimized by Chamberlain (10), which is herein referred to as conventional CDM (cCDM) and is currently used for specialized *F. tularensis* studies. Most of the information about amino acid metabolism in hypervirulent A.I SCHU S4 has been derived from *in silico* genome analyses. These assessments predicted the absence or incomplete synthesis pathways for at least six amino acids, specifically arginine, cysteine, histidine, lysine, methionine, and tyrosine (11–13). Moreover, most of the information pertaining to amino acid metabolism in *F. tularensis* is based on studies with attenuated LVS (14, 15). Although type B LVS is predicted to share similar amino acid auxotrophies with hypervirulent A.I SCHU S4, differences have been identified in tyrosine and polyamine biosynthesis pathways (13, 16). Therefore, a better understanding of hypervirulent SCHU S4 metabolism is needed.

Cultivation of *F. tularensis* in brain heart infusion broth (BHI) has been determined to recapitulate gene expression by this intracellular pathogen in infected macrophages, the primary replication niche (17). BHI provides critical factors, such as amino acids and polyamines, which derive from enzymatic digestions of animal brains and hearts (18, 19). All living organisms require polyamines for fitness and acquire these critical metabolites via the catabolism of several amino acids, uptake, and/or *de novo* synthesis, but the mechanisms that regulate polyamine homeostasis are ill defined (20–22). We previously revealed that only the *F. tularensis* A.I clade can synthesize the polyamines agmatine, putrescine, and spermidine *de novo*, which requires the procurement of both arginine and methionine from an external source (16). Based on our findings, we hypothesized that polyamine metabolism contributes to the fitness of hypervirulent A.I SCHU S4.

In the current study, we identified important differences and similarities pertaining to the metabolism of polyamine precursors and polyamines in *F. tularensis* SCHU S4 and LVS and their isogenic spermidine synthase SpeE mutant (Δ*speE*). Addition of spermidine to CDM not containing any polyamines (CDM-PA) promoted substantially faster growth by SCHU S4 and SCHU S4 Δ*speE* but caused biphasic growth by LVS and LVS Δ*speE*, with concentrations above 0.1 mM being inhibitory. In CDM-PA, SCHU S4 Δ*speE* grew in a diauxic manner, which was alleviated by methylthioadenosine, adenosine, or adenine supplementation, revealing a crucial link between *de novo* polyamine biosynthesis and adenine salvage. Comparisons between optical density readings and associated viable counts for *F. tularensis* SCHU S4 and SCHU S4 Δ*speE* confirmed that SpeE contributes to the fitness of this intracellular pathogen by increasing the replication rate and tolerance to stress when nutrient availability is low and bacterial cell densities are high. Together, these findings reveal previously unknown variable traits for the two most studied *F. tularensis* strains, specifically hypervirulent SCHU S4 and attenuated LVS.

## RESULTS

### Disparities in the ability of *F. tularensis* subpopulations to synthesize polyamines

**Fig 1:**
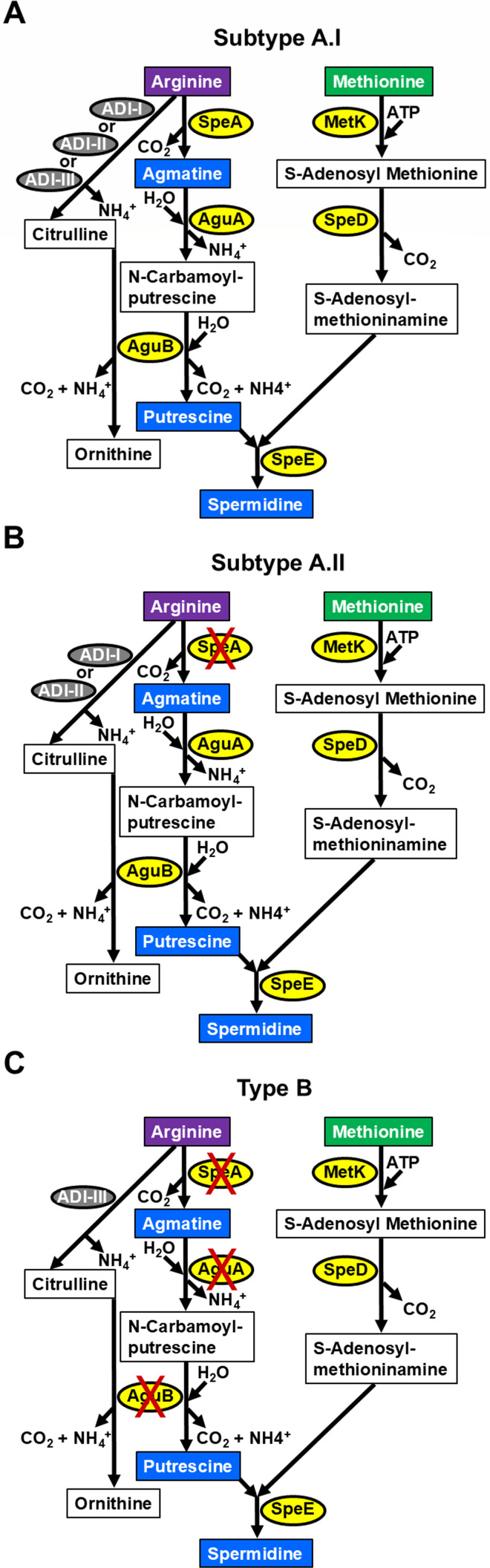
Polyamine biosynthesis pathway in *F. tularensis* subtype A.I, subtype A.II, and type B clades. Both arginine and methionine are essential amino acids in *F. tularensis* that are required for *de novo* polyamine synthesis, along with the enzymes (yellow ovals) arginine decarboxylase SpeA, agmatine deiminase AguA, N-carbamoylputrescine amidase AguB, methionine adenosyltransferase (also known as S-adenosylmethionine synthetase) MetK, S-adenosylmethionine decarboxylase SpeD, and spermidine synthase SpeE. (**A**) Only *F. tularensis* A.I strains (e.g., SCHU S4) contain intact genes that encode all six enzymes required for the endogenous synthesis of the polyamines agmatine, putrescine, and spermidine (blue rectangles). These enzymes in SCHU S4 include SpeA (FTT_0432), AguA (FTT_0434), AguB (FTT_0435), MetK (FTT_0149), SpeD (FTT_0430), and SpeE (FTT_0431). (**B**) In *F. tularensis* subtype A.II strains (e.g., WY96-3418), the gene encoding SpeA is truncated, and (**C**) in *F. tularensis* type B strains (e.g., LVS), the genes encoding SpeA, AguA, and AguB are disrupted due to the insertion of a transposase gene and subsequent genomic rearrangements. Also shown are arginine deiminases (ADI, gray ovals) that catabolize arginine to citrulline and ammonia. *F. tularensis* subtype A.I strains encode three ADIs, including a 183-, 304-, and 307-residue enzyme (ADI-I, ADI-II, and ADI-III), subtype A.II strains encode the 183- and 304-amino acid ADIs (ADI-I and ADI-II), and type B strains encode only the 307-residue enzyme (ADI-III).

We previously reported on the genomic and transcriptomic differences in the metabolic enzymes required for *de novo* polyamine biosynthesis pathways in the *F. tularensis* A.I, A.II, and B clades (16). More specifically, only the hypervirulent subtype A.I strains can synthesize the polyamines agmatine, putrescine, and spermidine *de novo*, which requires the metabolism of both arginine and methionine (Fig. 1A). In A.II strains, the initial gene in arginine catabolism for subsequent polyamine biosynthesis encodes arginine decarboxylase SpeA and is prematurely truncated (Fig. 1B). Type B strains lack the first three enzymes in arginine catabolism for *de novo* polyamine synthesis due to genomic rearrangements that disrupt the genes encoding arginine decarboxylase SpeA, agmatine deiminase AguA, and N-carbamoylputrescine amidase AguB (Fig. 1C). However, genes encoding methionine adenosyltransferase MetK and S-adenosyl methionine (SAM) decarboxylase SpeD (previously annotated as SpeH) in the methionine metabolic branch for spermidine synthesis, as well as spermidine synthase SpeE, are intact in all *F. tularensis* subpopulations (Fig. 1).

A primary sequence alignment of the SpeE, the final enzyme required for spermidine synthase, showed four residue differences in this protein between representative strains from subtype A.I (SCHU S4 and MA00-2987), subtype A.II (WY96-3418 and WY-00W4114), and type B (LVS and FSC200) (Fig. S1). Two amino acid changes were conservative, and the other two dissimilarities were nonconservative within each subpopulation. Although this enzyme is conserved in all *F. tularensis* strains and identical within each clade, the functional contribution of SpeE to fitness in SCHU S4 and LVS was unknown and evaluated in this study.

Preliminary proteome analysis of *F. tularensis* subtype A.I SCHU S4 and MA00-2987, subtype A.II WY96-3418, and type B LVS grown to mid-exponential phase in BHI supplemented with cysteine revealed high levels of MetK in these strains (MassIVE accession number MSV000091876). Evaluation of SCHU S4 and LVS proteomes by others also reported high levels of MetK (23), while another global study confirmed the essentiality of this enzyme in *F. tularensis* SCHU S4 (24).

Arginine deiminase (ADI) is another enzyme involved in the catabolism of arginine in *F. tularensis* that converts this essential amino acid to citrulline and ammonia. *F. tularensis* subtype A.I encodes three ADI, including a 183, 304, and 307 residue enzyme (ADI-I, ADI-II, and ADI-III), subtype A.II encodes a 183 and 304 amino acid ADI (ADI-I and ADI-II), and type B encodes only the 307 residue ADI-III enzyme (Fig. 1). The clade-specific differences in ADI, SpeA, AguA, and AguB content, along with the regulation of these enzymes, undoubtedly influence the levels of the associated metabolites.

### Differential effects of polyamine precursors and polyamines on *F. tularensis* growth

**Fig 2:**
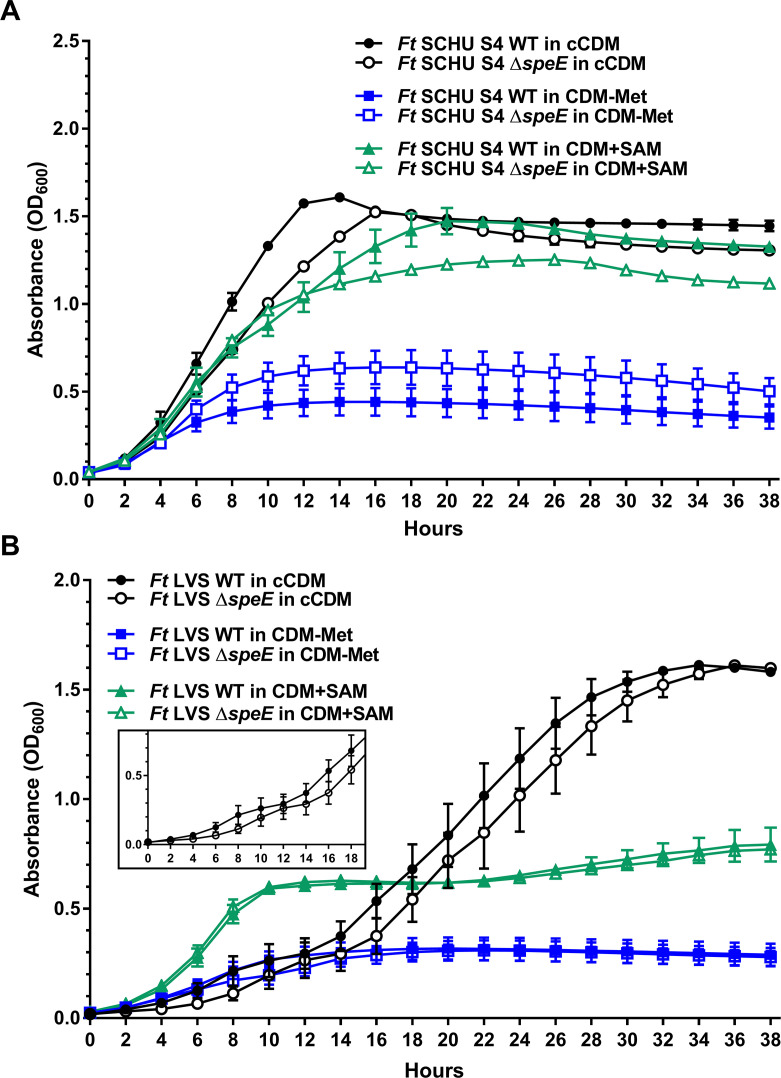
Growth of *F. tularensis* subtype A.I SCHU S4 and type B LVS and their isogenic Δ*speE* mutant in conventional chemically defined medium, which contains spermidine and methionine for comparison to growth in chemically defined medium without methionine or with S-adenosyl methionine. The growth of *F. tularensis* (*Ft*) (**A**) SCHU S4 and (**B**) LVS wild-type strains (WT, filled symbols) and isogenic Δ*speE* mutants (unfilled symbols) in conventional chemically defined medium that typically contains spermidine and methionine (cCDM, black circles), modified CDM without methionine (CDM-Met, blue squares), and modified CDM with S-adenosyl methionine (CDM+SAM, green triangles) are shown. Insert in Fig. 2B shows diauxic growth of LVS and LVS Δ*speE* in cCDM. Data represent the mean ± SEM error bars of three biological replicates in each of three independent experiments.

To evaluate and compare growth requirements, *F. tularensis* SCHU S4 and LVS strains, along with the isogenic Δ*speE* mutants, were grown in Chamberlain’s CDM that conventionally contains 0.1 mM spermidine (cCDM) for comparison to CDM that contained one or several altered components. Although SCHU S4 consistently grew faster and to a higher cell density than SCHU S4 Δ*speE* in cCDM (Fig. 2A) based on optical density at 600 nm (OD_600_), the growth rate of both A.I strains was approximately twofold faster than LVS and LVS Δ*speE* (Fig. 2). SCHU S4 and SCHU S4 Δ*speE* reached the highest cell density after 14 h (OD_600_ of 1.6) and 16 h (OD_600_ of 1.5) of growth in cCDM, respectively (Fig. 2A). In contrast, both LVS and LVS Δ*speE* grew in a diauxic manner in cCDM, reaching maximum cell density (OD_600_ of 1.55) after 34 and 36 h, respectively (Fig. 2B, insert). Together, these results indicated that spermidine synthase SpeE in SCHU S4 contributes to higher overall growth rate in cCDM compared to SCHU S4 Δ*speE* and LVS, and that both LVS and LVS Δ*speE* exhibit biphasic growth in this medium.

When methionine was omitted from cCDM (CDM-Met), growth was minimal for all four *F. tularensis* strains (Fig. 2). SCHU S4 Δ*speE*, however, reached a slightly higher cell density (OD_600_ of 0.6) relative to SCHU S4 (OD_600_ of 0.5), with both LVS and LVS Δ*speE* only attaining a maximum OD_600_ of 0.3 (Fig. 2). These results were expected due to the predicted auxotrophy of *F. tularensis* for this amino acid based on Kyoto Encyclopedia of Genes and Genomes (KEGG) analyses and studies associated with the development of a CDM to grow this fastidious pathogen (8–10).

Next, the effect of SAM on *F. tularensis* growth was evaluated, since this multifunctional metabolite is a precursor for spermidine production and derives from methionine. These results showed that the overall growth of both SCHU S4 and SCHU S4 Δ*speE* was only slightly inhibited by the exogenous addition of SAM to cCDM (CDM+SAM) with these strains reaching a maximum OD_600_ of 1.5 and 1.2, respectively (Fig. 2A). In contrast, SAM stimulated the initial 10 h of growth for both LVS and LVS Δ*speE* to a similar extent, but then growth plateaued and only reached a maximum OD_600_ of 0.75 (Fig. 2B). These data indicated that SCHU S4 is better adapted to excess SAM relative to LVS.

**Fig 3:**
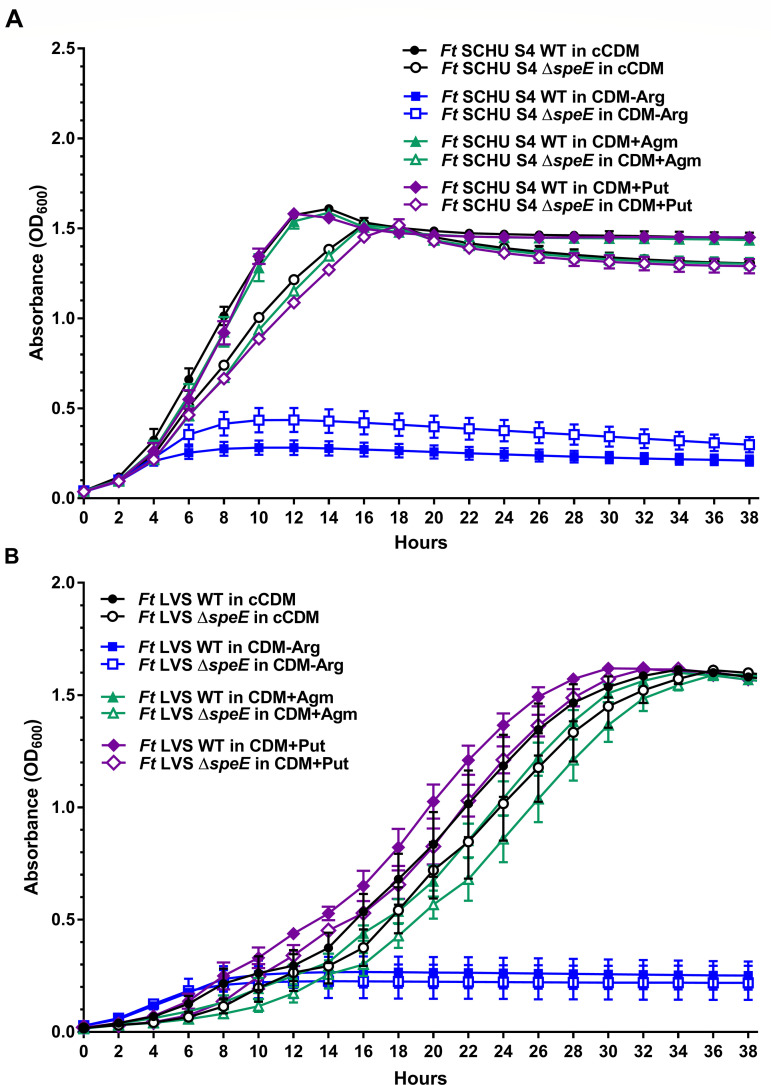
Growth of *F. tularensis* subtype A.I SCHU S4 and type B LVS and their isogenic Δ*speE* mutant in conventional chemically defined medium that typically contains spermidine and arginine for comparison to growth in modified chemically defined medium without arginine or with agmatine or putrescine. The growth of *F. tularensis* (*Ft*) (**A**) SCHU S4 and (**B**) LVS wild-type strains (WT, filled symbols) and isogenic Δ*speE* mutants (unfilled symbols) in conventional chemically defined medium that typically contains spermidine and arginine (cCDM, black circles), modified CDM without arginine (CDM-Arg, blue squares), modified CDM with agmatine (CDM+Agm, green triangles), and modified CDM with putrescine (CDM+Put, purple diamonds) is shown. Data represent the mean ± SEM error bars of three biological replicates in each of three independent experiments.

Like methionine, the absence of arginine in cCDM (CDM-Arg) did not support substantial growth by SCHU S4 and LVS nor the isogenic Δ*speE* mutants (Fig. 3). These results were anticipated since *F. tularensis* genomes do not encode an intact biosynthetic pathway for this essential amino acid. Again, SCHU S4 Δ*speE* reached a slightly higher maximum cell density relative to SCHU S4, albeit low (OD_600_ of 0.4 versus 0.3), while both LVS strains only reached a maximum OD_600_ of 0.25 (Fig. 3). Collectively, these results suggested that *de novo* spermidine biosynthesis by SCHU S4 may hinder growth when methionine or arginine levels are insufficient, since these essential amino acids are needed for other cellular functions.

Agmatine and putrescine were added separately to cCDM (CDM+Agm or CDM+Put, respectively) to determine if either of these polyamines promote *F. tularensis* growth. Neither SCHU S4 nor SCHU S4 Δ*speE* growth was altered when cCDM contained agmatine or putrescine relative to their growth in cCDM without either of these polyamines (Fig. 3A). These data supported the premise that these A.I strains can synthesize agmatine and putrescine (Fig. 1A), and that exogenous supplementation of these polyamines does not provide a fitness advantage. For LVS and LVS Δ*speE*, addition of agmatine slightly inhibited growth, while putrescine supplementation only modestly enhanced the growth rate (Fig. 3B). These results correlated with the inability of type B strains to catabolize agmatine and synthesize putrescine due to the absence of functional AguA and AguB enzymes, respectively (Fig. 1C).

### Spermidine uptake enhances *F. tularensis* growth

**Fig 4:**
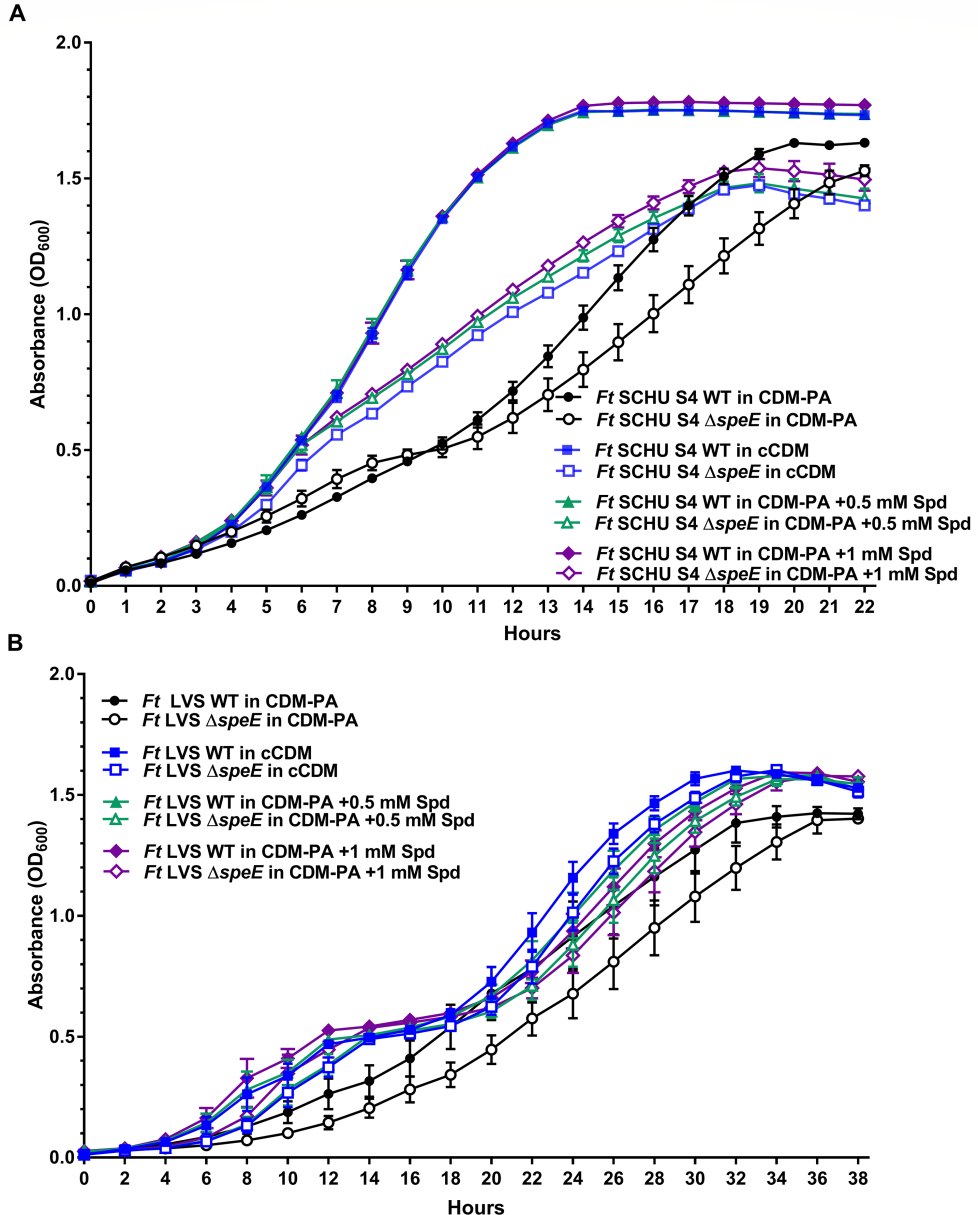
Spermidine import by *F. tularensis* SCHU S4 and LVS and their isogenic Δ*speE* mutant. The growth of *F. tularensis* (*Ft*) (**A**) SCHU S4 and (**B**) LVS wild-type strains (WT, filled symbols) and isogenic Δ*speE* mutants (unfilled symbols) in modified chemically defined medium without any polyamines (CDM-PA, black circles), conventional CDM that typically contains 0.1 mM spermidine (cCDM) (blue squares), CDM with 0.5 mM spermidine (CDM + 0.5 mM Spd, green triangles), and CDM with 1 mM spermidine (CDM + 1 mM Spd, purple diamonds) are shown. Data represent the mean ± SEM error bars of three biological replicates in each of three independent experiments.

To evaluate the contribution of spermidine to *F. tularensis* fitness, SCHU S4 and LVS and their associated Δ*speE* mutants were cultured in CDM without spermidine or any other polyamine (CDM-PA) for comparison to growth in cCDM, which typically contains 0.1 mM spermidine. In CDM-PA, the highest OD_600_ reached by SCHU S4 was 1.6 after 20 h, while SCHU S4 Δ*speE* grew similar to SCHU S4 for the initial 8 h but then entered a lag phase for 2.5 h, reaching an OD_600_ of 1.5 after 22 h (Fig. 4A). In cCDM that contains 0.1 mM spermidine, SCHU S4 and SCHU S4 Δ*speE* grew to a maximum OD_600_ of 1.75 after 14 h and an OD_600_ of 1.4 after 18 h, respectively, with spermidine addition eliminating the biphasic growth of SCHU S4 Δ*speE* that occurred in CDM-PA (Fig. 4A). Increasing the concentration of spermidine from 0.1 to 1 mM did not alter SCHU S4 growth but slightly augmented the growth rate of SCHU S4 Δ*speE* in a dose-dependent manner (Fig. 4A). At 3 mM spermidine, again no difference in growth rate was observed for SCHU S4, while SCHU S4 Δ*speE* overall cell density slightly increased relative to 0.1 mM of this triamine (Fig. S2). In addition, decreasing the concentration of spermidine to 0.02 mM was sufficient to promote optimum SCHU S4 growth (Fig. S3).

*F. tularensis* SCHU S4 and SCHU S4 Δ*speE* growth in cCDM containing 0.1 mM spermidine was next compared to growth in CDM in which spermidine was replaced with spermine or spermine phosphate at 0.1 or 0.5 mM. No difference in growth rate nor maximum cell density was observed for SCHU S4 nor SCHU S4 Δ*speE* when cultured with spermidine, spermine, or spermine phosphate at either concentration (Fig. S4A). In addition, including both spermidine and spermine at 0.1 mM did not further enhance the growth rate of SCHU S4 (Fig. S4B). Together, these results indicated that spermidine and spermine equally increase the growth rate of SCHU S4 and SCHU S4 Δ*speE* but did not rescue SCHU S4 Δ*speE*, indicating that other critical factors are required.

Neither *F. tularensis* LVS nor LVS Δ*speE* grew in a biphasic manner in CDM-PA but took 34 and 36 h, respectively, to reach a maximum OD_600_ of 1.4 (Fig. 4B). In cCDM that contains 0.1 mM spermidine, these LVS strains grew to slightly higher maximum OD_600_ of 1.55, but the addition of spermidine again caused diauxic growth and concentrations higher than 0.1 mM were slightly inhibitory (Fig. 4B). These results revealed that spermidine uptake and utilization are differentially regulated in these A.I and B strains. Furthermore, the promotion of substantially faster replication by SCHU S4 during *de novo* synthesis and/or import of spermidine suggests an adaptation to this polyamine for enhanced fitness.

**Fig 5:**
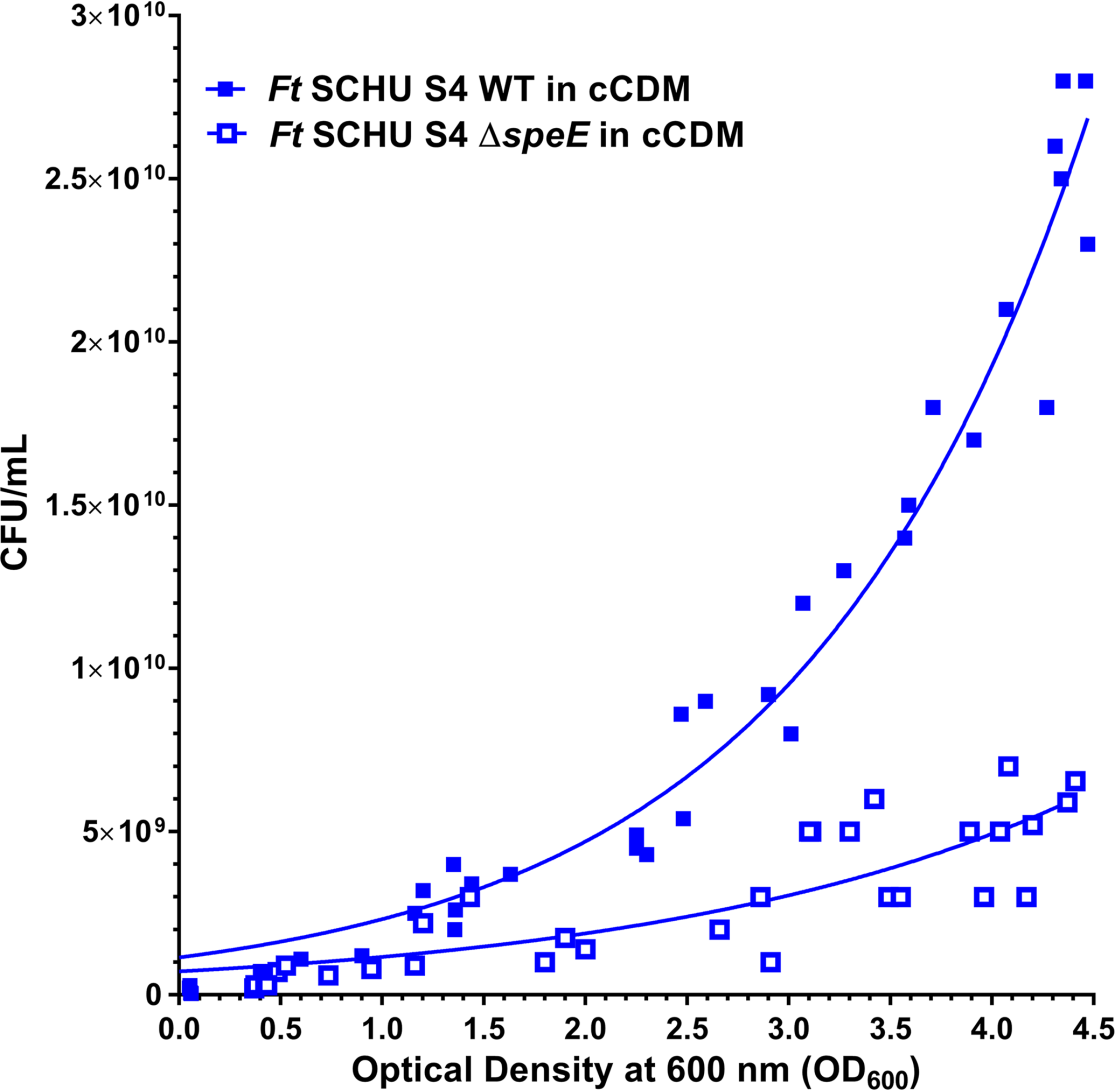
Standard curve of optical density at 600 nM versus viable CFU/mL for *F. tularensis* SCHU S4 and SCHU S4 Δ*speE* during growth. Shown is a comparison of optical density readings at 600 nm and the corresponding viable CFUs/mL for *F. tularensis* (*Ft*) SCHU S4 wild type (WT, filled squares) and SCHU S4 Δ*speE* (mutant, unfilled squares) during growth in conventional chemically defined medium that typically contains 0.1 mM spermidine (cCDM). Data represent three biological replicates in each of two independent experiments. Least squares (ordinary) fit with 95% confidence intervals for nonlinear regression is also shown.

To better understand the growth dynamics between *F. tularensis* SCHU S4 and SCHU S4 Δ*speE*, viable CFU/mL versus OD_600_ values were compared during growth in cCDM. These results showed that these strains have a unique relationship between CFU/mL and OD_600_ readings with SCHU S4 having a markedly higher number of viable cells at a similar OD measurement than SCHU S4 Δ*speE* (Fig. 5). These results verified that SCHU S4 replicates at a substantially faster rate than SCHU S4 Δ*speE* and suggested that mutant cells were larger in size than SCHU S4 due to cell division defects and/or that some mutant cells die but do not lyse.

Lag phase duration for *F. tularensis* SCHU S4 and SCHU S4 Δ*speE* grown in cCDM was determined to be 3.25 and 3.71 h, respectively (Table 1). In addition, assessment of growth rate and doubling time revealed that SCHU S4 grew exponentially after lag phase up to approximately 10 h, with growth continuing albeit more slowly up to at least 20 h when the experiment was terminated. In contrast, logarithmic growth for SCHU Δ*speE* only occurred from about 4 to 6 h with no detectable cell division after 14 h. Collectively, these data confirmed that SpeE serves a critical role for SCHU S4 fitness via increasing the replication rate and promoting survival during stressful conditions, such as decreased nutrient availability and high cell densities.

**TABLE 1:** *F. tularensis* SCHU S4 and SCHU S4 Δ*speE* lag phase duration, growth rate constants, and doubling times based on viable cells

*F. tularensis* strain*^a^*	Lag phase duration*^b^*	Hours of growth and time interval	Growth rate (µ)*^c^*	Doubling time (τ)*^c^*
SCHU S4 WT	3.25 h			
		2 h from 4 to 6 h	0.381 h^−1*^	1.82 h
		2 h from 6 to 8 h	0.312 h^−1^	2.22 h
		2 h from 8 to 10 h	0.306 h^−1^	2.27 h
		2 h from 10 to 12 h	0.248 h^−1^	2.79 h
		2 h from 12 to 14 h	0.187 h^−1^	3.71 h
		2 h from 14 to 16 h	0.159 h^−1^	4.36 h
		2 h from 16 to 18 h	0.153 h^−1^	4.53 h
		2 h from 18 to 20 h	0.122 h^−1^	5.68 h
SCHU S4 Δ*speE*	3.71 h			
		2 h from 4 to 6 h	0.367 h^−1*^	1.89 h
		2 h from 6 to 8 h	0.132 h^−1^	5.25 h
		2 h from 8 to 10 h	0.113 h^−1^	6.13 h
		2 h from 10 to 12 h	0.089 h^−1^	7.79 h
		2 h from 12 to 14 h	0.059 h^−1^	11.75 h
		2 h from 14 to 16 h	–*^d^*	–*^d^*
		2 h from 16 to 18 h	–*^d^*	–*^d^*
		2 h from 18 to 20 h	–*^d^*	–*^d^*

^*a*^ Strains were grown in flasks containing conventional chemically defined medium with shaking at 37°C.

^*b*^ Lag phase duration was defined as the time interval before the bacterial population begins exponential growth at the maximum growth rate (µ_max_), which is denoted with an asterisk (*), and was determined using the equation t_lag_ = t_1_ − ln(N_1 _– N_0_)/µ_max_, in which N_1_ is the number of viable bacteria at the beginning of exponential phase and N_0_ is the number of viable bacteria at the end of lag phase.

^*c*^ The growth rate constant (µ) per hour was determined using the equation µ = ln (N_t2_/N_t0_)/τ, and doubling time (τ) was determined using the equation τ = ln(2)/µ.

^*d*^ No detectable increase in viable cell counts.

### Metabolic profiling of polyamine precursor levels in *F. tularensis*

**Fig 6:**
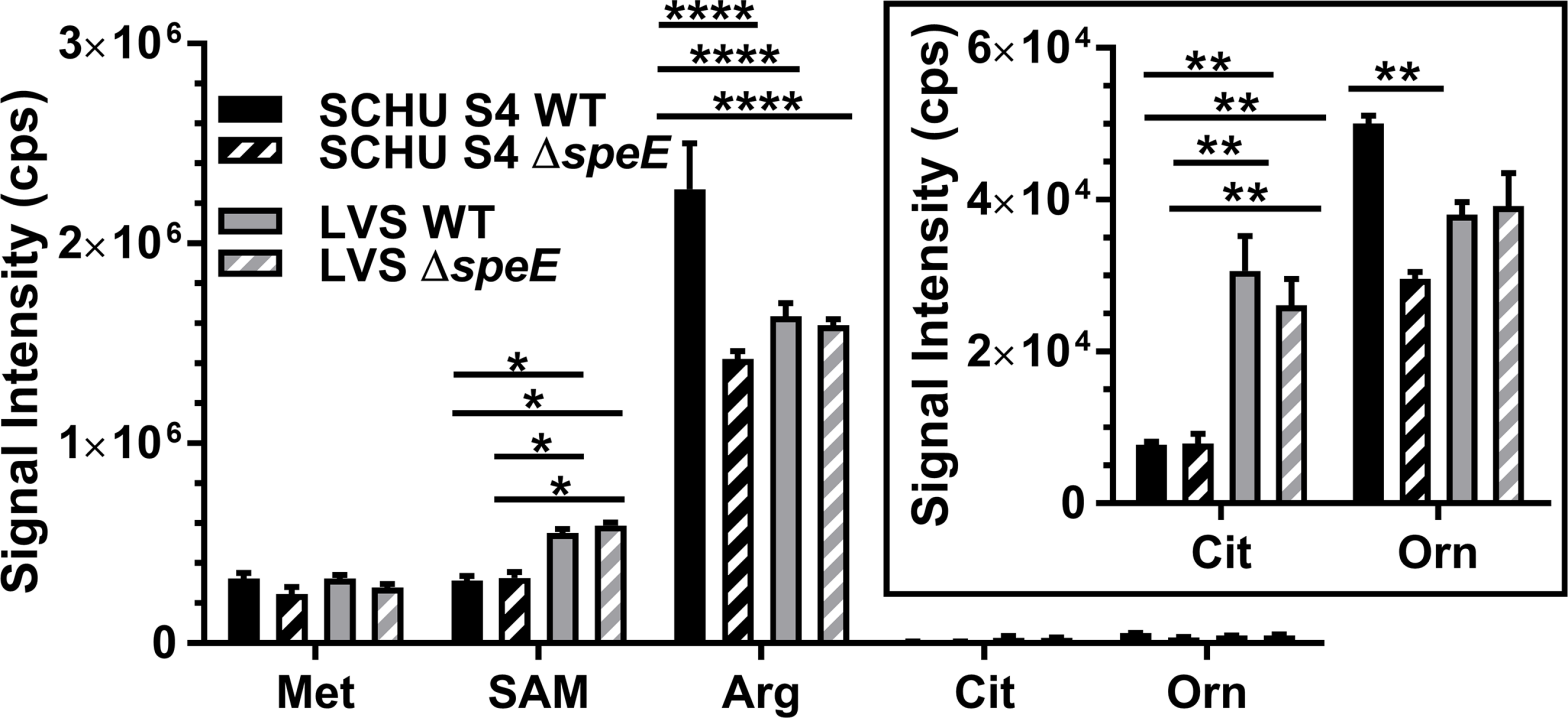
Metabolic profiles of methionine and arginine, along with other polyamine precursors in *F. tularensis* subtype A.I SCHU S4 and type B LVS and their isogenic ∆*speE* mutants during exponential growth in BHI. Shown is a comparison of relative methionine (Met), S-adenosyl methionine (SAM), arginine (Arg), citrulline (Cit), and ornithine (Orn) abundance in wild-type (WT) strains and isogenic ∆*speE* mutants. Insert shows the relative levels of citrulline (Cit) and ornithine (Orn). Mean ± SEM are shown for triplicate samples in two independent experiments. Data were analyzed using two-way ANOVA with multiple comparisons and Tukey’s post hoc or unpaired *t*-test with a two-tailed analysis, as appropriate. Only significant differences (*P* < 0.05) are denoted. *, *P* < 0.05; **, *P* < 0.01; ****, *P* < 0.0001.

To assess the abundance of polyamine precursor substrates in *F. tularensis*, targeted metabolic profiling was performed using ultra-performance liquid chromatography coupled with tandem mass spectrometry (UPLC-MS/MS). For these evaluations, mid-log phase *F. tularensis* SCHU S4 and LVS, along with the associated Δ*speE* mutants, were grown in BHI and then processed as described in the methods section. These assessments revealed that cellular methionine levels were similar in these strains (Fig. 6), which was also the case when grown in CDM-PA (Fig. S5). These data corroborated with our previous study that showed no difference in mRNA abundance in different *F. tularensis* strains for the methionine importer *metNQI* operon and *metK* regardless of external methionine concentrations (16). Together, these results showed that methionine metabolism in *F. tularensis* is highly conserved regardless of the presence or absence of SpeE.

Examination of SAM abundance revealed slightly higher levels of this critical factor in LVS and LVS Δ*speE* relative to SCHU S4 and SCHU S4 Δ*speE* when grown in BHI (Fig. 6). A similar result was obtained when these strains were grown in CDM-PA (Fig. S5). Furthermore, the concentration of this multifunctional metabolite was equivalent between SCHU S4 and SCHU S4 Δ*speE* and between LVS and LVS Δ*speE* mutants, indicating tight and clade-specific regulation of SAM abundance (Fig. 6). The lower levels of SAM in SCHU S4 compared to LVS may explain why SCHU S4 can tolerate higher external levels of this metabolite than LVS, as was shown in Fig. 2, and suggest more effective utilization of this metabolite. This trait is important during an intracellular infection since activated macrophages generate SAM due to increased metabolic activity (25).

Interestingly, SCHU S4 had a significantly higher abundance of arginine than SCHU S4 Δ*speE*, LVS, and LVS Δ*speE,* whether grown in BHI (Fig. 6) or CDM-PA (Fig. S5). This result was not anticipated since A.I strains have three ADIs in contrast to type B strains with only one ADI, and since arginine decarboxylase SpeA is intact in only A.I strains (Fig. 1). We also previously reported that transcriptional expression of the arginine importer ArgP was not altered in SCHU S4 and SCHU S4 Δ*speE* despite altered arginine concentrations in CDM (16). These findings collectively suggest that there are additional importers of this essential amino acid and/or that the mechanisms regulating internal arginine levels are positively influenced by *de novo* spermidine synthesis in SCHU S4.

Citrulline abundance in the SCHU S4 strains was approximately threefold lower in comparison to the LVS strains (Fig. 6, insert). This data were expected since *F. tularensis* type A strains have citrullinase activity due to bifunctional AguB, which can catabolize both N-carbamoylputrescine and citrulline while type B strains lack this enzyme (Fig. 1), and since citrullinase activity is used to distinguish these two subspecies (26, 27). In addition, ornithine levels were higher in SCHU S4 relative to SCHU S4 Δ*speE* (Fig. 6, insert), which suggests that *de novo* spermidine synthesis promotes AguB activity.

Next, we sought to determine if the absence of SpeE in *F. tularensis* SCHU S4 Δ*speE* increases medium acidification due to feedback inhibition of upstream enzymes that generate ammonium ions during arginine catabolism (i.e., AguA, AguB, and ADI). During the initial 10 h, growth was slow and similar for both strains, while the pH of the medium increased from 6.45 to 6.8 (Fig. S6). After 10 h, the growth rate of SCHU S4 increased substantially, which continued for an additional 16 h, whereas the growth rate of SCHU S4 Δ*speE* remained steady but slower (Fig. S6A). During the exponential growth of SCHU S4, the average pH of the medium was 6.75, while the average pH of SCHU S4 Δ*speE* medium was slightly higher at 6.85 (Fig. S6B). After 26 h for SCHU S4 and 36 h for SCHU S4 Δ*speE*, a maximum OD_600_ of 3 and 2.6 was attained, respectively. SCHU S4 medium remained at pH 6.75 for 4 h while in the stationary phase, but then the pH increased to 7.2 by 36 h, at which time some cell lysis was noted. In comparison, the pH of the SCHU S4 Δ*speE* medium gradually increased to a similar pH by 36 h. Together, these findings revealed that the ablation of SpeE in SCHU S4 does not substantially contribute to the acidification of CDM-PA, and that media alkalinization contributes to *F. tularensis* growth arrest, which supports an earlier study (8).

### Metabolic profiling of polyamine levels in *F. tularensis*

**Fig 7:**
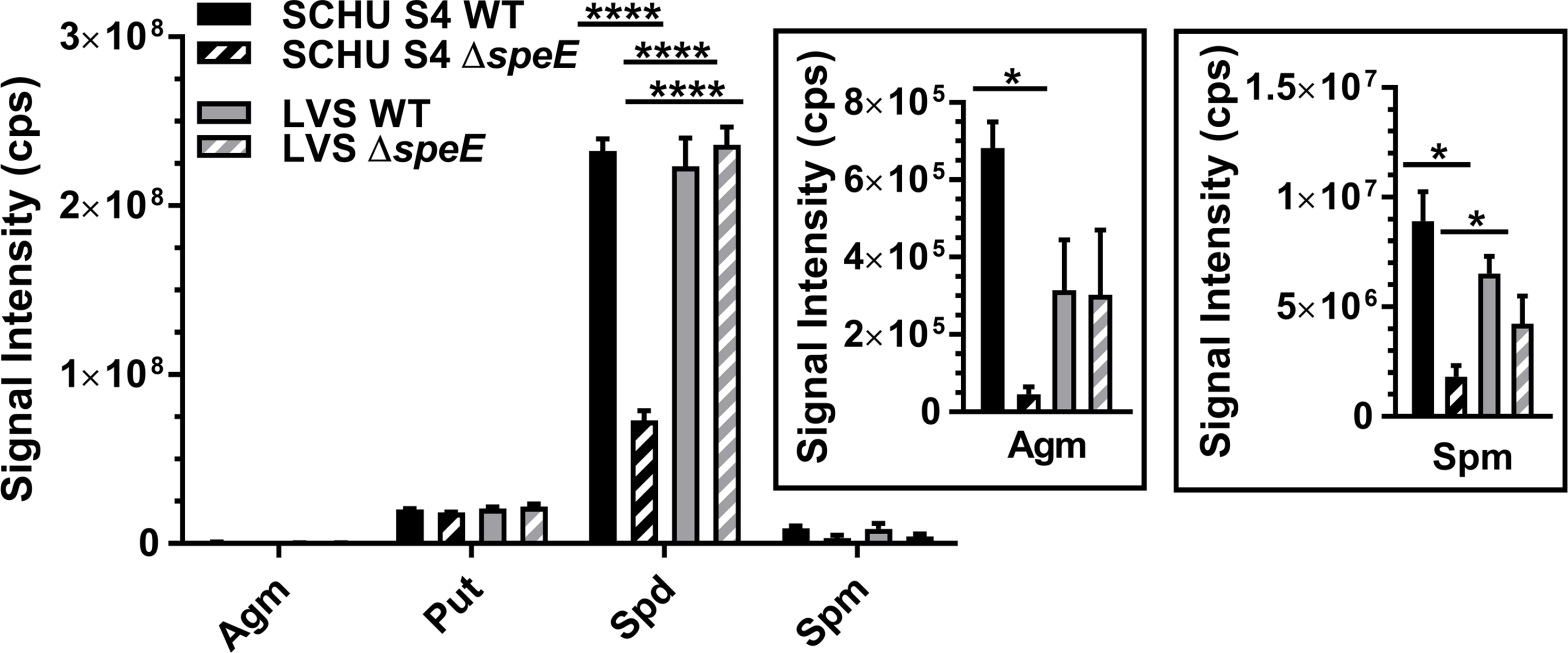
Metabolic profiles of polyamine levels in *F. tularensis* subtype A.I SCHU S4 and type B LVS and their isogenic mutants during exponential growth in BHI. Shown is a comparison of relative agmatine (Agm), putrescine (Put), spermidine (Spd), and spermine (Spm) abundance in wild-type (WT) strains and isogenic ∆*speE* mutants. Insert shows the relative levels of agmatine (Agm) and spermine (Spm). Mean ± SEM are shown for triplicate samples in two independent experiments. Data were analyzed using two-way ANOVA with multiple comparisons and Tukey’s post hoc tests or unpaired *t*-test with a two-tailed analysis, as appropriate. Only significant differences (*P* < 0.05) are denoted. *, *P* < 0.05; ****, *P* < 0.0001.

Targeted LC-MS/MS analysis of polyamines in *F. tularensis* SCHU S4, SCHU S4 Δ*speE*, LVS, and LVS Δ*speE* cultured to mid-log growth phase in BHI was evaluated. These data showed that SCHU S4 had a slightly higher abundance of agmatine than SCHU S4 Δ*speE*, whereas the levels of this polyamine were similar in LVS and LVS Δ*speE* (Fig. 7, insert). These results suggested that the absence of SpeE in SCHU S4 Δ*speE* negatively affects SpeA activity and/or agmatine import, while the LVS strains were unaltered.

Putrescine levels in *F. tularensis* SCHU S4, SCHU S4 Δ*speE*, LVS, and LVS Δ*speE* were similar (Fig. 7). Although the SCHU S4 strains can synthesize putrescine *de novo*, type B strains, such as LVS, must import this polyamine. Therefore, the equivalent levels of putrescine in these four strains indicated a notable role for this diamine in *F. tularensis* that is highly regulated.

Although spermidine levels were equivalent in SCHU S4, LVS, and LVS Δ*speE*, this polyamine was significantly reduced in SCHU S4 Δ*speE* (Fig. 7). These results demonstrated a greater reliance on SpeE by SCHU S4 for spermidine production than LVS, and that both synthesis and import of this critical triamine are impaired in SCHU S4 Δ*speE*.

Since eukaryotes produce spermine, a polyamine shown to protect against oxidative stress (21), and since *F. tularensis* is an intracellular pathogen, import of this polyamine from BHI by SCHU S4, SCHU S4 Δ*speE*, LVS, and LVS Δ*speE* was evaluated. These data revealed that eukaryotic spermine is imported by these *F. tularensis* strains. However, SCHU S4 Δ*speE* had the lowest abundance of this tetramine (Fig. 7), suggesting that importation is negatively affected in the absence of *de novo* spermidine synthesis.

### Methylthioadenosine, adenine, and adenosine alleviate diauxic growth by *F. tularensis* SCHU S4 Δ*speE*

**Fig 8:**
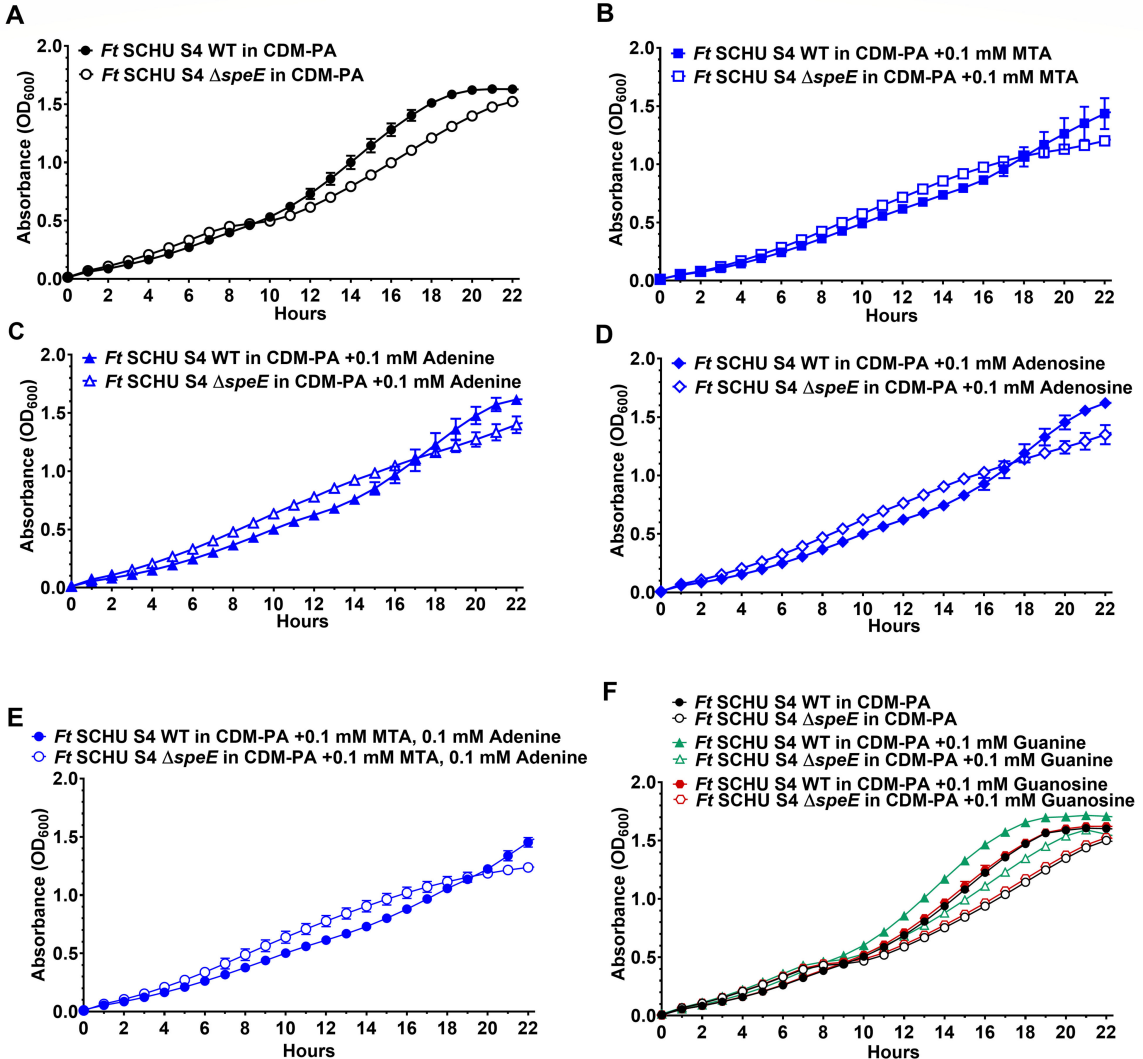
Diauxic growth of *F. tularensis* SCHU S4 Δ*speE* in chemically defined medium without spermidine is alleviated by methylthioadenosine, adenine, or adenosine supplementation. Growth of *F. tularensis* (*Ft*) SCHU S4 wild type (WT, filled symbols) and the isogenic Δ*speE* mutant (unfilled symbols) in (**A**) chemically defined medium without polyamines (CDM-PA, black circles), (**B**) CDM-PA with 0.1 mM methylthioadenosine (CDM-PA + 0.1 mM MTA, blue squares), (**C**) CDM-PA with 0.1 mM adenine (CDM-PA + 0.1 mM adenine, blue triangles), (**D**) CDM-PA with 0.1 mM adenosine (CDM-PA + 0.1 mM adenosine, blue diamonds), (**E**) CDM-PA with 0.1 mM MTA and 0.1 mM adenine (CDM-PA + 0.1 mM MTA, 0.1 mM adenine, blue circles), and (**F**) CDM-PA (CDM-PA, black circles), CDM-PA with 0.1 mM guanine (CDM-PA + 0.1 mM guanine, green triangles), and CDM-PA with 0.1 mM guanosine (CDM-PA + 0.1 mM guanosine, red hexagons) are shown. Data represent the mean ± SEM error bars of three biological replicates in each of three independent experiments.

Spermidine supplementation to CDM-PA eliminated biphasic growth and enhanced the replication rate of *F. tularensis* SCHU S4 Δ*speE* but did not rescue this mutant (Fig. 4A). Therefore, different amino acids, peptide-containing components, and other factors at various concentrations were supplemented to CDM-PA for subsequent growth assessment of this mutant. Of these evaluations, only the addition of yeast extract rescued the SCHU S4 Δ*speE* mutant (Fig. S7). Since nucleic acid content in yeast extract is high (28), we hypothesized that perhaps methylthioadenosine (MTA), a byproduct of *de novo* spermidine biosynthesis, may rescue SCHU S4 Δ*speE*. We also evaluated the potential rescue of SCHU S4 Δ*speE* by adenine and adenosine supplementation, as well as other bases and their associated nucleoside at various concentrations. These analyses importantly revealed that only MTA, adenine, or adenosine at 0.1 mM alleviated the diauxic growth of SCHU S4 Δ*speE* (Fig. 8). When 0.1 mM MTA and 0.1 mM adenine were both added to CDM-PA, the partial rescue of SCHU S4 Δ*speE* was extended for an additional 2 h (Fig. 8E). However, the inclusion of spermidine with adenine and/or MTA made the partial rescue unnoticeable due to the considerable increase in growth rate promoted by this polyamine (Fig. S7). Furthermore, the other bases or nucleosides evaluated had no effect on growth, except for guanine. Guanine enhanced the growth rate of both SCHU S4 and SCHU S4 Δ*speE* but did not rescue the mutant, while guanosine had no effect on the growth of either strain (Fig. 8F). These data collectively indicated that the endogenous production of MTA, adenine, and adenosine by SCHU S4 provides more fitness than the importation of these factors, while exogenous spermidine substantially enhances the replication rate of both SCHU S4 and SCHU S4 Δ*speE*. Therefore, since only yeast extract fully rescued SCHU S4 Δ*speE*, additional factors within this component are required to overcome the fitness benefits of *de novo* spermidine synthesis in SCHU S4.

### Spermidine biosynthesis by *F. tularensis* SCHU S4 is linked to adenine but not methionine recycling

**Fig 9:**
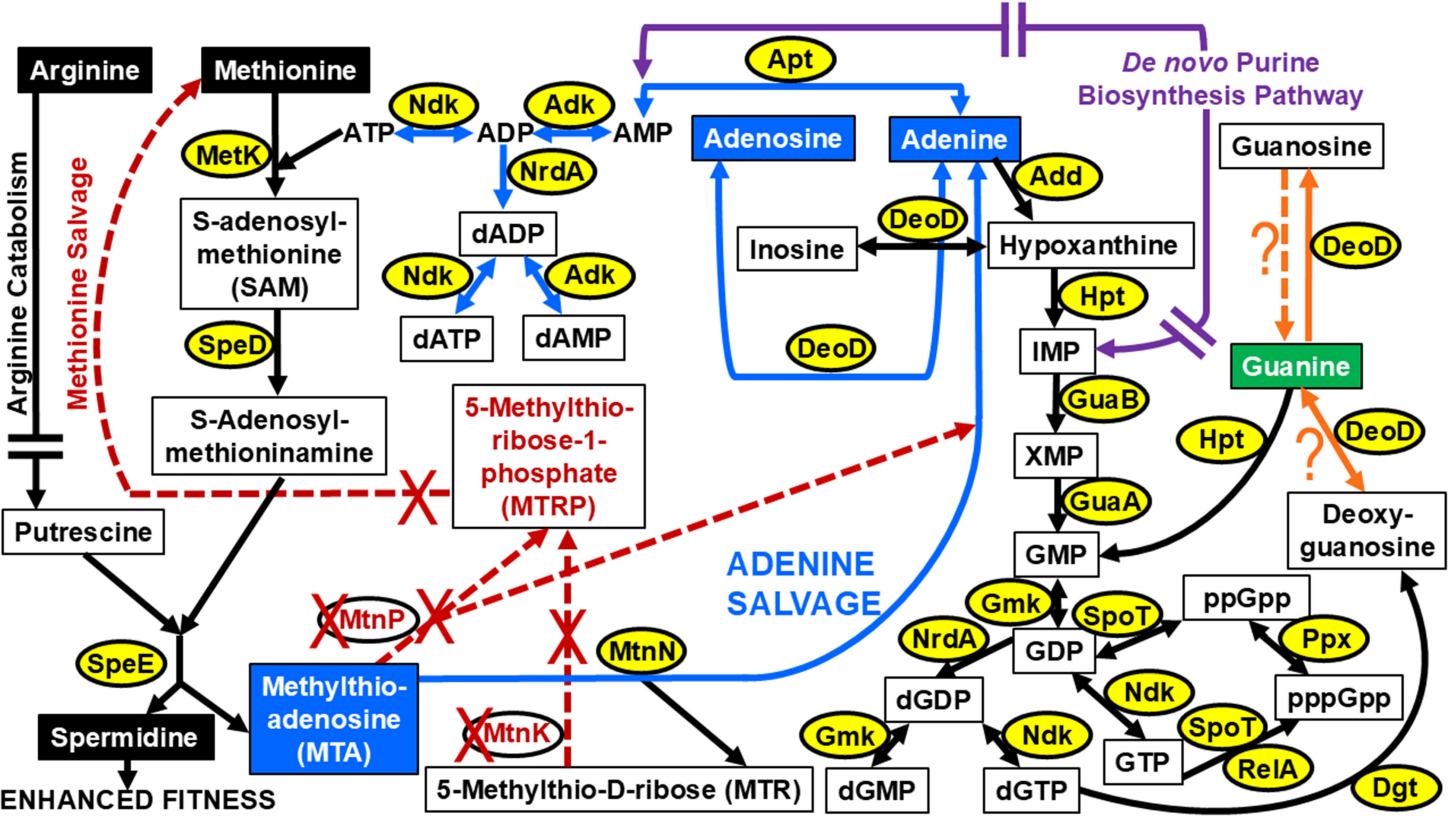
*De novo* spermidine biosynthesis in *F. tularensis* A.I SCHU S4 contributes to adenine salvage and fitness but not methionine recycling. SCHU S4 enzymes involved in methionine metabolism for adenine recycling include S-adenosylmethionine synthetase MetK (EC 2.5.1.6, FTT_0149c), S-adenosylmethionine decarboxylase SpeD (EC 4.1.1.50, FTT_0430), spermidine synthase SpeE (EC 2.5.1.16, FTT_0431), and methylthioadenosine (MTA) nucleosidase MtnN (EC 3.2.2.9, FTT_0397). Enzymes absent in the *F. tularensis* genome for subsequent methionine salvage include MTA phosphorylase MtnP (EC 2.4.2.28, pseudogene FTT_0608) and 5-methylthio-D-ribose (MTR) kinase MtnK (EC 2.7.1.100). Newer and revised annotations for SCHU S4 indicate that FTT_0608 is a pseudogene, so the associated pathways that are shown in KEGG for this enzyme have not been updated. An enzyme synthesizing guanine from guanosine is absent or inhibited in SCHU S4, as was shown in Fig. 8F, and see Fig. 1A for SCHU S4 enzymes involved in arginine catabolism in the *de novo* polyamine biosynthesis pathway. Other enzymes shown include nucleoside diphosphate kinase Ndk (EC 2.7.4.6, FTT_0373c), adenylate kinase Adk (EC 2.7.4.3, FTT_1161), ribonucleoside-diphosphate reductase NrdA (EC 1.17.4.1, FTT_0534c), adenine phosphoribosyltransferase Apt (EC 2.4.2.7, FTT_0078), purine nucleoside phosphorylase DeoD (EC 2.4.2.1, FTT_0766), adenine deaminase Add (EC 3.5.4.2, FTT_0939c), hypoxanthine phosphoribosyltransferase Hpt (EC 2.4.2.8, FTT_0205), IMP dehydrogenase GuaB (EC 1.1.1.205, FTT_1317c), GMP synthase GuaA (EC 6.3.5.2, FTT_1019c), guanylate kinase Gmk (EC 2.7.4.8, FTT_1470c), dGTPase Dgt (EC 3.1.5.1, FTT_0720c), (p)ppGpp synthase/GTP diphosphokinase/guanosine-3′,5′-bis(diphosphate) 3′-diphosphatase SpoT (EC 3.1.7.2/EC 2.7.6.5, FTT_0808), GTP pyrophosphokinase RelA (EC 2.7.6.5, FTT_1508c), and exopolyphosphatase Ppx (EC 3.6.1.11/EC 3.6.1.40, FTT_1444c). Enzymes shown with red text denote the absence of the gene encoding this protein in the SCHU S4 genome, and red arrows with a dashed line and “X” indicate the absence of these reactions. Orange arrows with a dashed or solid line and adjacent “?” signify that DeoD substrate specificity for guanine-based compounds and activity requires further study.

KEGG pathways associated with *de novo* spermidine and MTA production in *F. tularensis* SCHU S4 indicated that MTA nucleosidase MtnN (EC 3.2.2.9, FTT_0397) metabolizes MTA to produce adenine, which importantly salvages this purine (Fig. 9). Adenine provides a substrate for AMP or can be converted to adenosine, both of which are reversible reactions. AMP can then be converted to ADP for subsequent ATP or dADP production, and adenosine can serve as a substrate for inosine synthesis. Inosine can then be converted to hypoxanthine for the synthesis of guanine-based compounds (Fig. 9). The results shown in Fig. 8A through E supported these predicted metabolic pathways.

Based on KEGG analysis of purine biosynthesis in *F. tularensis* SCHU S4, purine phosphorylase DeoD (EC 2.4.2.1, FTT_0766) is required for the reversible production of adenine to adenosine, inosine to hypoxanthine, and guanine to deoxyguanosine. However, an enzyme that synthesizes guanine from guanosine appears to be lacking or is inhibited in this pathogen, which concurs with the result that guanine but not guanosine promoted faster growth by SCHU S4 and SCHU S4 Δ*speE* (Fig. 8F). Based on these findings, guanine-based compounds may not serve as a substrate for DeoD (Fig. 9). If this is the case, the reversible production of guanine to deoxyguanosine may not occur, which is in opposition to the role of DeoD shown in the KEGG pathway for SCHU S4, and hypoxanthine phosphoribosyltransferase Hpt (EC 2.4.2.8, FTT_0205) may solely convert guanine to other essential guanine-based compounds (Fig. 9). Therefore, further study is needed to evaluate DeoD activity and determine if guanine-based compounds can even serve as a substrate for this enzyme in *F. tularensis* SCHU S4.

In addition to adenine production, MtnN can also catabolize MTA to produce 5-methylthio-D-ribose (MTR). However, *F. tularensis* does not contain a gene encoding MTR kinase MtnK (EC 2.7.1.100) to salvage methionine, nor is a functional MTA phosphorylase MtnP (EC 2.4.2.28) present to convert MTA to 5-methylthio-ribose-1-phosphate for methionine recycling (Fig. 9). Nevertheless, these data collectively demonstrated that not only does *de novo* spermidine synthesis promote rapid replication by SCHU S4 but that the byproduct MTA salvages adenine, reducing the need for *de novo* purine biosynthesis that requires 10 enzymes in 13 catabolic reactions (Fig. 9).

## DISCUSSION

We showed that considerable differences exist between the *F. tularensis* subpopulations in the pathways associated with arginine and polyamine metabolism, whereas methionine metabolism is highly conserved. This latter feature is supported by the similar cellular levels of this essential amino acid and the lack of a methionine salvage pathway during spermidine synthesis, affirming the importance of methionine import in *F. tularensis*. We also revealed that hypervirulent *F. tularensis* SCHU S4 has evolved to depend on both *de novo* biosynthesis and uptake of spermidine to promote fitness, in contrast to LVS. Although *de novo* synthesis of spermidine in *F. tularensis* SCHU S4 promotes faster replication than SCHU S4 Δ*speE* regardless of the absence or presence of this polyamine in CDM, spermidine uptake promotes even higher fitness, which could be due in part to the accumulation of the dead-end byproduct MTR.

Others have shown that SCHU S4 Δ*aguB* was impaired for intramacrophage growth, and mice intranasally infected with this mutant had a significantly reduced bacterial burden (29), indicating that arginine metabolism and polyamine synthesis by this A.I strain contributes to virulence. Moreover, the high levels of the essential amino acid arginine in SCHU S4 may promote citrulline catabolism by AguB to ornithine for concomitant ornithine export and arginine import, but this will require further study.

Growth curves reflect the overall physiological state of a given strain’s population over time, that is highly dependent on nutrient availability, waste product accumulation, and other factors. However, since optical density does not measure viability, a comparison between optical density and the associated viable CFU/mL was evaluated for *F. tularensis* SCHU S4 and SCHU S4 Δ*speE*. The similar optical density readings between SCHU S4 and SCHU S4 Δ*speE* at later stages of growth, but a substantially lower number of viable mutant cells compared to wild type, indicated that in the absence of SpeE, functional defects occur in the replication machinery and/or survival mechanism. Future experiments utilizing electron microscopy may provide additional evidence that SCHU S4 Δ*speE* is defective in cellular division after growth commences, which results in a larger cell size compared to wild type, as was suggested by the viable CFUs and OD reading comparisons. Nonetheless, these findings confirm that SpeE serves an important fitness role in hypervirulent SCHU S4 that contributes to rapid replication and stress tolerance, especially when nutrient availability is low and bacterial cell densities are high.

We importantly confirmed that *de novo* biosynthesis of spermidine by *F. tularensis* SCHU S4 salvages adenine via the catabolism of MTA, a byproduct in this pathway. Adenine is a major energy carrier and a precursor for the synthesis of nucleotides and nucleotide cofactors, including ATP and guanine-based compounds. In *F. tularensis*, *de novo* synthesis of purines requires PurF, PurCD, PurN, PurT, PurL, PurM, PurK, PurE, PurB, PurH, and PurA, which are located at five different loci. Therefore, MTA nucleosidase MtnN (EC 3.2.2.9) provides a critical function in *F. tularensis* SCHU S4 and other A.I strains by converting MTA to adenine, conserving cellular energy (Fig. 9).

Polyamine structure and charge influence their electrostatic interactions with various cellular factors and processes (e.g., replication, transcription, translation, ion transport, and membrane dynamics) (21, 22). The complex and content-dependent metabolism of polyamines, which varies between organisms, hinders a comprehensive understanding of their specific mode of action (20, 30–32). Nevertheless, our data indicate that *de novo* spermidine synthesis by SCHU S4 contributes to the fitness of this intracellular pathogen by salvaging adenine, promoting faster cell division, and increasing stress tolerance when nutrient availability is low and bacterial cell densities are high, while exogenous spermidine or spermine further enhances the replication rate. These previously unknown traits suggest an adaptation by A.I strains to an intracellular environment with spermine signaling the intracellular milieu of the infected host cell. Accordingly, notable studies by Nau and associates reported the identification of spermine-responsive genes in *F. tularensis* that are involved in virulence (33, 34).

In summary, the variable environments and hosts encountered by zoonotic and facultative intracellular pathogens, such as *F. tularensis,* certainly require nutritional plasticity. The disparities in SCHU S4 and LVS metabolism, as was revealed in this study, demonstrate that caution is warranted when generalizing results that were obtained from a single *F. tularensis* clade or strain. Moreover, these findings underscore the important role of arginine and polyamine metabolism in the adaptation of *F. tularensis* A.I strains to an intracellular milieu. Studies are currently underway to evaluate the contribution of these cationic metabolites to the successful persistence of *F. tularensis* SCHU S4 and other hypervirulent A.I strains during an intracellular infection.

## MATERIALS AND METHODS

### Bacterial strains and culturing

*F. tularensis* SCHU S4, MA00-2987, WY96-3418, and LVS were obtained from the Biodefense and Emerging Infections Research Resources Repository. Select agent *F. tularensis* strains were transferred to the University of Nebraska Medical Center following the requirements of the Federal Select Agent Program as outlined in the Animal and Plant Health Inspection Service/CDC Form 2. Manipulation of viable culture material was performed by authorized individuals within a biosafety level 3 laboratory certified for select agent work using laboratory biosafety criteria, according to requirements of the Federal Select Agent Program. For UPLC-MS/MS metabolite analyses to be performed outside of this facility, *F. tularensis* preparations were inactivated with ethanol, followed by sterility testing.

For each experiment, *F. tularensis* strains were initially cultured from a master glycerol stock onto chocolate agar plates and then grown in BHI supplemented with cysteine or CDM at 37°C, as previously described (16). Conventional cCDM was prepared as previously described (10) or was modified as denoted for the relevant experiments. SAM was added to CDM at the same concentration as methionine in cCDM, whereas agmatine and putrescine were added at the same concentration as spermidine and spermine in cCDM. Growth curves were obtained utilizing a Tecan Spark platform or flasks in which OD_600_ readings were recorded every hour or every 2 h, respectively.

### Construction and complementation of *F. tularensis* Δ*speE* mutants

In-frame and markerless *speE* deletion mutants were generated in *F. tularensis* SCHU S4 and LVS using methods developed by Horzempa et al. (35). Details pertaining to the construction and transcomplementation of these mutants were described and shown in our preceding study (16).

### Interpretation of *F. tularensis* growth

A standard curve of CFU/mL versus OD_600_ was obtained for *F. tularensis* SCHU S4 and SCHU S4 Δ*speE*, to better understand the growth dynamics between these strains. For these experiments, these strains were cultured in flasks containing cCDM with shaking at 37°C, in which both OD_600_ readings and the corresponding viable cell counts were obtained. For each OD_600_ reading, appropriate dilutions of the culture were plated on chocolate agar plates to acquire the number of viable bacteria. Plates were incubated at 37°C for 5 days, and then CFU/mL were enumerated for both SCHU S4 and SCHU S4 Δ*speE* for comparison to the associated OD_600_ reading.

Lag phase duration was defined as the time interval before the bacterial population begins exponential growth at the maximum growth rate (µ_max_), and was determined using the equation t_lag_ = t_1_ − ln(N_1_ − N_0_)/µ_max_, in which N_1_ is the number of viable bacteria at the beginning of exponential phase and N_0_ is the number of viable bacteria at the end of lag phase. The growth rate constant (µ) and doubling time (τ) were also determined. The growth rate constant (µ) per hour was determined using the equation µ = ln (N_t2_/N_t0_)/τ, and doubling time (τ) was determined using the equation τ = ln(2)/µ. For these equations, N_t_ is the number of bacteria present at a specified time, N_0_ is the starting number of bacteria, t is the time interval during which growth is measured, and ln(2) is the natural logarithm of 2. The growth rate constant (µ) was depicted in units of time (i.e., h^−1^), and the doubling time (τ) was the time required for the population to double in number.

### Metabolite processing and targeted mass spectrometry

*F. tularensis* was grown to mid-log growth phase in BHI or CDM-PA, washed with cold 0.9% sodium chloride, and then resuspended in ice-cold 60% ethanol that contained internal standards. Cells were further processed for UPLC-MS/MS analyses, as described in the Supplemental materials.

The concentration of non-derivatized and derivatized metabolites was determined from calibration curves of genuine standards with known concentrations and plotted against their corresponding peak areas. For analysis of underivatized polyamine precursors, the internal standards included 20 uniformly ^13^C/^15^N-labeled canonical amino acids (Cambridge Isotope Laboratories). For the derivatized polyamines, the internal standards were deuterated putrescine (CAS #284665-22-1, Sigma) and deuterated spermidine (CAS #1173019-26-5, Sigma). The compound-specific parameters, such as MRM parameters, collision energy, and retention time for each metabolite, including the internal standards, are shown in Table 2.

**TABLE 2:** UPLC-MS/MS metabolite-specific parameters for the UPLC-MS/MS analyses

UPLC-MS/MS metabolite-specific parameters					
Sr number	Component name	Mass information	Polarity	Rt (min)	Collision energy (V)
1	Agmatine	439.3/91.1	+	10.9	71.0
2	Putrescine	397.1/155.1	+	11.6	34.0
3	Spermidine	608.3/226.3	+	12.7	37.0
4	Spermine	819.3/212.1	+	13.3	35.0
5	Putrescine_d8 (internal standard)	405.0/155.1	+	11.6	34.0
6	Spermidine_d8 (internal standard)	616.2/234.0	+	12.6	37.0
7	SAM	399.1/250.1	+	1.55	20.8
8	Methionine	150.2/104.0	+	2.01	15.0
9	Ornithine	133.1/70.1	+	1.53	17.4
10	Arginine	173.0/131.0	−	1.58	−19.1
11	Citrulline	174.0/131.0	−	1.79	−19.0
12	Arginine-^13^C_6_^15^N_4_ (internal standard)	185.1/74.9	+	6.68	25.0
13	Arginine-^13^C_6_^15^N_4_ (internal standard)	182.9/138.1	−	6.67	−18.1
14	Methionine-^13^C_6_^15^N_4_ (internal standard)	156.2/60.3	+	3.04	27.0

### Statistical analysis

Data are presented as means ± standard error of mean (±SEM) and are representative of duplicates or triplicates in not less than two independent experiments. Data from experiments with one variable were analyzed using Student’s *t*-test. Data from experiments with multiple variables were analyzed utilizing two-way ANOVA and Tukey’s post-test. Statistical significance was set at a *P* value of <0.05, and statistical analyses were performed using GraphPad Prism.

## Data Availability

Proteome data sets for *F. tularensis* SCHU S4, MA00-2987, WY96-3418, and LVS were submitted to Mass Spectrometry Interactive Virtual Environment (MassIVE), a full member of the consortium ProteomeXchange, and were assigned MassIVE accession number MSV000091876. Metabolome data sets for *F. tularensis* are provided upon request.

## SUPPLEMENTAL MATERIAL

Supplemental materials jb.00616-25-s0001.pdf Supplemental methods and Figures S1 to S7.

ASM does not own the copyrights to Supplemental Material that may be linked to, or accessed through, an article. The authors have granted ASM a non-exclusive, world-wide license to publish the Supplemental Material files. Please contact the corresponding author directly for reuse.
