# Supplementary material for: Spermidine biosynthesis by hypervirulent Francisella tularensis promotes fitness and salvages adenine
Source: J Bacteriol. 2026 Apr 22;208(5):e00616-25. doi: 10.1128/jb.00616-25 (PMC13192272; doi:10.1128/jb.00616-25)
Supplement: Supplemental materials — Supplemental methods and Figures S1 to S7. [file jb.00616-25-s0001.pdf]

SUPPLEMENTAL MATERIAL

Spermidine Biosynthesis by Hypervirulent *Francisella tularensis* Promotes Fitness and Salvages Adenine

Yinshi Yue[a*], Dhanajay Shinde[a*], Robert Moore II[b], Yangsheng Yu[a], Vinai Chittezham Thomas[a], Tomáš Helikar[b], and Marilynn A. Larson[a]#

[a]Department of Pathology, Microbiology, and Immunology, University of Nebraska Medical Center, Omaha, Nebraska, USA
[b]Department of Biochemistry, University of Nebraska-Lincoln, Lincoln, Nebraska, USA

*Authors contributed equally.
#Address correspondence to Marilynn A. Larson, malarson@unmc.edu.

SUPPLEMENTAL METHODS

Processing of bacterial cells for liquid chromatography-tandem mass spectrometry analysis of metabolites

Bacterial cell suspensions in ice-cold 60% ethanol were lysed using 0.1 mm diameter glass beads in a FastPrep homogenizer (MP Biomedicals) with dry ice in the CoolPrep adapter. Lysates were then centrifuged, supernatants were collected, and then equally divided for underivatized polyamine precursor and derivatized polyamine processing. The samples were lyophilized and resuspended in an appropriate buffer. For underivatized polyamine precursor assessment, samples were resuspended in 10 mM ammonium acetate and 10 mM ammonium hydroxide at pH 8. For the evaluation of polyamines, samples were derivatized using tosyl chloride (Sigma), as previously described by Nalazek-Rudnicka K. and Wasik A. [Monatsh Chem (2017) 148:1685-1696]. The concentration of underivatized and derivatized metabolites in the samples were determined using targeted ultra-performance liquid chromatography coupled with tandem mass spectrometry (UPLC-MS/MS) analyses, as described below in the Supplemental Methods.

Targeted ultra-performance liquid chromatography-tandem mass spectrometry

Ultra-performance liquid chromatography coupled with tandem mass spectrometry (UPLC-MS/MS) analyses were performed using an Acquity *I*-Class UPLC coupled with a Sciex QTRAP 6500 Plus mass spectrometer (Waters Corporation). Non-derivatized and derivatized metabolites were separated on a 150 x 2.1 mm BEH C18 column with a 3 µm particle size (Waters Corporation) that was connected to a similar 20 x 2.1 mm guard column. Mobile phase A was composed of 0.1% formic acid in water, and mobile phase B was made up of 0.1% formic acid in acetonitrile. The following gradient program with a consistent flow rate of 0.20 mL/minute was applied: Isocratic hold 7% B for 3 minutes, linear gradient from 7% B to 35% B for 4 minutes, linear gradient from 35% B to 95% B for another 6 minutes, isocratic hold 95% B for 3 minutes

followed by column equilibration at 7% B for 5.9 minutes. Data was acquired in multiple-reaction mode (MRM) using polarity switching in positive and negative mode. Electrospray ionization (ESI) parameters were as follows: capillary voltage of 5.5 kV in positive mode and 4.5 kV in negative mode, curtain gas flow rate was 35 Au, gas 1 and gas 2 were set at 45 AU, the capillary temperature was 400°C, CAD gas was at medium level. MS instrument was tuned for declustering potential (DP) at 65V (+) and -65V (-), entrance potential (EP) at 10V (+) and -10V (-), and collision cell exit potential (CXP) at 10V (+) and -10V (-).

The nonderivatized metabolites such as SAM, methionine, ornithine, arginine, and citrulline were separated on a 150 × 2.1 mm ID XBridge Amide analytical column with a 1.7 µm particle size (Waters Corporation), using an Acquity I-Class ultraperformance liquid chromatography and a binary solvent system (Waters Corporation) that was infused at a flow rate of 0.3 mL/minute. A 20 × 2.1 mm ID guard XBridge Amide column with a 3.5 µm particle size (Waters Corporation) was connected in front of analytical column. Mobile phase A was composed of 10 mM ammonium acetate and 10 mM ammonium hydroxide containing 5% acetonitrile in LC-MS grade water with a pH of 8.0, which was obtained using glacial acetic acid. Mobile phase B was 100% LC-MS grade acetonitrile. The UHPLC pumps were operated in gradient mode. The amide column was maintained at 40°C and the autosampler was maintained at 5°C during data acquisition. MS data was acquired using the MRM-mode on a Sciex QTRAP 6500 Plus mass spectrometer (Waters Corporation).

The injection volume for all samples was 5 µL. The concentration of non-derivatized and derivatized metabolites was determined from calibration curves of genuine standards with known concentrations and plotted against their corresponding peak areas. For analysis of underivatized polyamine precursors, the internal standards included 20 uniformly $^{13}C/^{15}N$-labeled canonical amino acids (Cambridge Isotope Laboratories). For the derivatized polyamines, the internal standards were deuterated putrescine (CAS #284665-22-1, Sigma) and deuterated spermidine (CAS #1173019-26-5, Sigma). The compound-specific parameters such as MRM parameters, collision energy, and retention time for each metabolite including the internal standards are shown in Table 2.

FIGURE S1 Protein alignment of spermidine synthase protein (SpeE) from representative strains from each *F. tularensis* subpopulation. These *F. tularensis* strains included subtype A.I strains SCHU S4, MA00-2987, subtype A.II strains WY96-3418 and WY-00W4114, and type B strains FSC200 and LVS. Clustal Omega (version 1.2.4) was used to produce the protein alignment shown.

```
LVS             MIANINNKKIFHETLYHSYHQSIAISEILYEHKTDYQHLVIFNNPIFGNVMVLDGIVQTT  60
FSC200          MIANINNKKIFHETLYHSYHQSIAISEILYEHKTDYQHLVIFNNPIFGNVMVLDGIVQTT  60
SCHU S4         MIANINNKKIFHETLYHSYHQSIAISEILYEHKTDYQHLVIFNNPIFGNVMVLDGIVQTT  60
MA00-2987       MIANINNKKIFHETLYHSYHQSIAISEILYEHKTDYQHLVIFNNPIFGNVMVLDGIVQTT  60
WY96-3418       MIANINNKKIFHETLYHSYHQSIAISEILYEHKTDYQHLVIFNNPIFGNVMVLDGIVQTT  60
WY-00W4114      MIANINNKKIFHETLYHSYHQSIAISEILYEHKTDYQHLVIFNNPIFGNVMVLDGIVQTT  60
                ************************************************************

LVS             EKDEFIYHEMLVHVPVIAHGNVKNILIIGGGDGGMLREALSHKAVEFVTLVEIDQAVIDM  120
FSC200          EKDEFIYHEMLVHVPVIAHGNVKNILIIGGGDGGMLREALSHKAVEFVTLVEIDQAVIDM  120
SCHU S4         EKDEFIYHEMLVHVPVIAHGNVKNILIIGGGDGGMLREALSHKAVESVTLVEIDQAVIDM  120
MA00-2987       EKDEFIYHEMLVHVPVIAHGNVKNILIIGGGDGGMLREALSHKAVESVTLVEIDQAVIDM  120
WY96-3418       EKDEFIYHEMLVHVPVIAHGNVKNILIIGGGDGGMLREALSHKAVESVTLVEIDQAVIDM  120
WY-00W4114      EKDEFIYHEMLVHVPVIAHGNVKNILIIGGGDGGMLREALSHKAVESVTLVEIDQAVIDM  120
                ********************************************** *************

LVS             CQEYFPGHSKGAFDHPKAKIVIQDGCEFVKNPPRKYDLIICDSTDPIGPGEVLFTSKFYK  180
FSC200          CQEYFPGHSKGAFDHPKAKIVIQDGCEFVKNPPRKYDLIICDSTDPIGPGEVLFTSKFYK  180
SCHU S4         CQEYFPGHSKGAFDHPKAKIVIQDGCEFVKNPPRKYDLIICDSTDPIGPGEVLFTSKFYK  180
MA00-2987       CQEYFPGHSKGAFDHPKAKIVIQDGCEFVKNPPRKYDLIICDSTDPIGPGEVLFTSKFYK  180
WY96-3418       CQEYFPGHSKGAFDHPKAKIVIQDGCEFVKNPPRKYDLIICDSTDPIGPGEVLFTSKFYK  180
WY-00W4114      CQEYFPGHSKGAFDHPKAKIVIQDGCEFVKNPPRKYDLIICDSTDPIGPGEVLFTSKFYK  180
                ************************************************************

LVS             DCKEALNPGGIMVTQNGVIYFQIDELKKTLERFEPLYKDVSFYTAAVPTYVGGSMAFGWG  240
FSC200          DCKEALNPGGIMVTQNGVIYFQIDELKKTLERFEPLYKDVSFYTAAVPTYVGGSMAFGWG  240
SCHU S4         DCKEAINPGGIMVTQNGVIYFQIDELKKTLERFEPLYKDVSFYTAAVPTYVGGSMAFGWG  240
MA00-2987       DCKEAINPGGIMVTQNGVIYFQIDELKKTLERFEPLYKDVSFYTAAVPTYVGGSMAFGWG  240
WY96-3418       DCKEALNPDGIMVTQNGVIYFQIDELKKTLERFEPLYKDVSFYTAAVPTYVGGSMAFGWG  240
WY-00W4114      DCKEALNPDGIMVTQNGVIYFQIDELKKTLERFEPLYKDVSFYTAAVPTYVGGSMAFGWG  240
                *****:**.***************************************************

LVS             TDELSYRNHDIQVIAQRFLKSGIKTKYYNPAIHIAAFALPQYVIDTLKK  289
FSC200          TDELSYRNHDIQVIAQRFLKSGIKTKYYNPAIHIAAFALPQYVIDTLKK  289
SCHU S4         TDELSYRNHDIQVIAQRFLKSGIKTKYYNPAIHIAAFALPQYVIDTLKK  289
MA00-2987       TDELSYRNHDIQVIAQRFLKSGIKTKYYNPAIHIAAFALPQYVIDTLKK  289
WY96-3418       TDELSYRNHDIQVIAQRFLKSGIKTRYYNPAIHIAAFALPQYVIDTLKK  289
WY-00W4114      TDELSYRNHDIQVIAQRFLKSGIKTRYYNPAIHIAAFALPQYVIDTLKK  289
                *************************:***********************
```

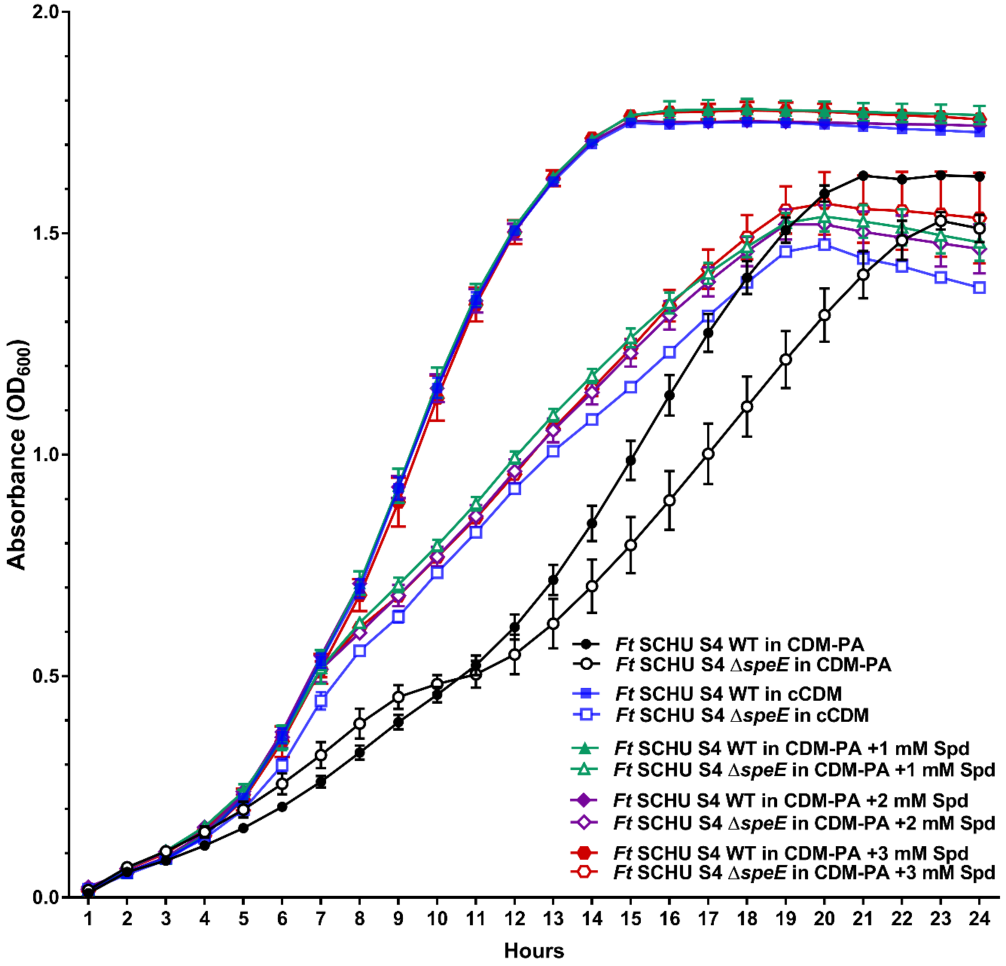

FIGURE S2 Growth of *F. tularensis* SCHU S4 and isogenic Δ*speE* mutant in modified chemically defined medium without any polyamines or with high amounts of spermidine. Shown is the growth of *F. tularensis* (*Ft*) SCHU S4 wildtype (WT, filled symbols) and the isogenic Δ*speE* mutant (unfilled symbols) in chemically defined medium without any polyamines (CDM-PA) or with a high concentration of spermidine (Spd) as denoted. Data represents the mean ± SEM error bars of three biological replicates in each of three independent experiments.

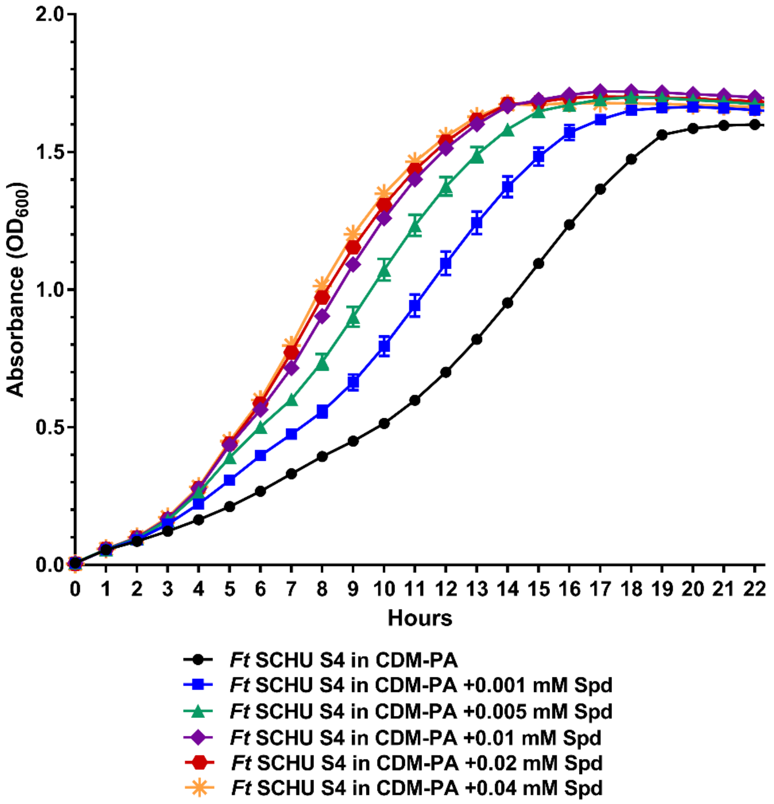

FIGURE S3 Growth of *F. tularensis* SCHU S4 in chemically defined medium without or with low amounts of spermidine. Shown is the growth of SCHU S4 in chemically defined medium without any polyamines (CDM-PA) or with a low concentration of spermidine (Spd) as denoted. Data represents the mean ± SEM error bars of three biological replicates in each of three independent experiments.

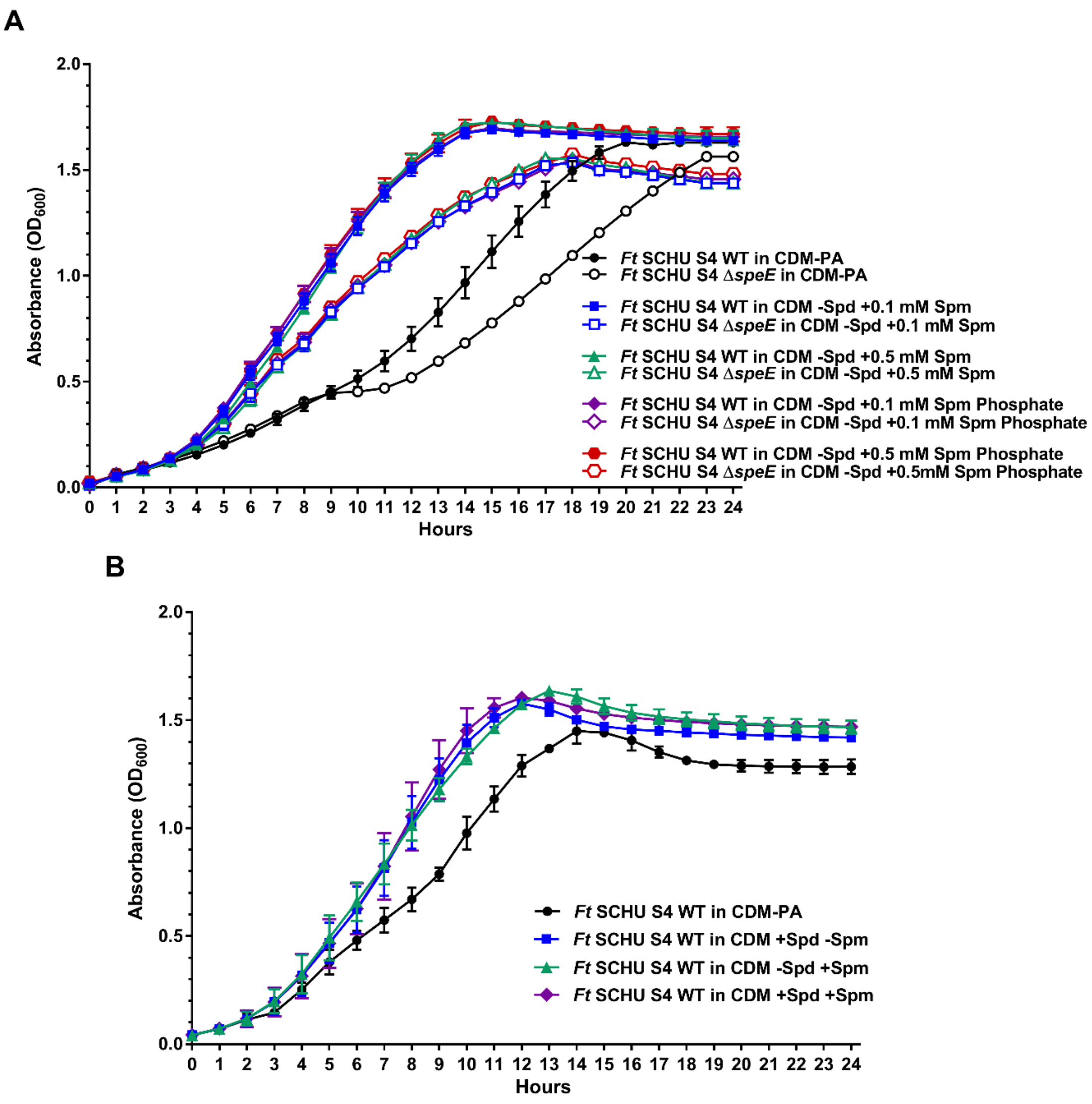

FIGURE S4 Growth of *F. tularensis* SCHU S4 and isogenic Δ*speE* mutant in chemically defined medium without or with spermidine, spermine, and spermine phosphate. **(A)** Shown is the growth of SCHU S4 wildtype (WT, filled symbols) and the SCHU S4 Δ*speE* mutant (unfilled symbols) in chemically defined medium without any polyamines (CDM-PA, black circles), and CDM without spermidine but with 0.1 mM spermine (CDM -Spd +0.1 mM Spm, blue squares), without spermidine but with 0.5 mM spermine (CDM -Spd +0.5 mM Spm, green triangles), without spermidine but with 0.1 mM spermine phosphate (CDM -Spd +0.1 mM Spm Phosphate, purple diamonds), and without spermidine but with 0.5 mM spermine phosphate (CDM -Spd +0.5 mM Spm Phosphate, red hexagons). **(B)** Shown is the growth of SCHU S4 wildtype (WT) in chemically defined medium without any polyamines (CDM-PA, black circles), with 0.1 mM spermidine and no spermine (CDM +Spd -Spm, blue squares), without spermidine but with 0.1 mM spermine (CDM -Spd +Spm, green triangles), and with both 0.1 mM spermidine and 0.1 mM spermine (CDM +Spd +Spm, purple diamonds). Data represents the mean ± SEM error bars of two to four biological replicates in each of two independent experiments.

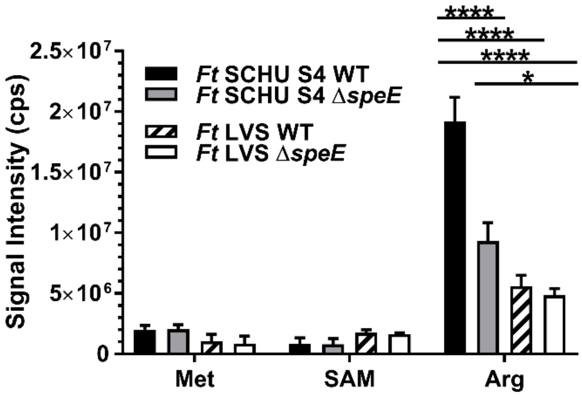

FIGURE S5 Endogenous levels of methionine, SAM, and arginine in *F. tularensis* SCHU S4 and LVS and their isogenic Δ*speE* mutants during exponential growth in chemically defined medium without any polyamines. Shown is a comparison of relative methionine (Met), S-adenosyl methionine (SAM), and arginine (Arg) levels in *F. tularensis* (*Ft*) SCHU S4 and LVS wildtype (WT) and isogenic Δ*speE* mutant strains. Mean ± SEM are shown for duplicate samples in two independent experiments. Data were analyzed using two-way ANOVA with multiple comparisons and Tukey's post hoc tests. Only significant differences ($P < 0.05$) are denoted. *, $P < 0.05$; ****, $P < 0.0001$.

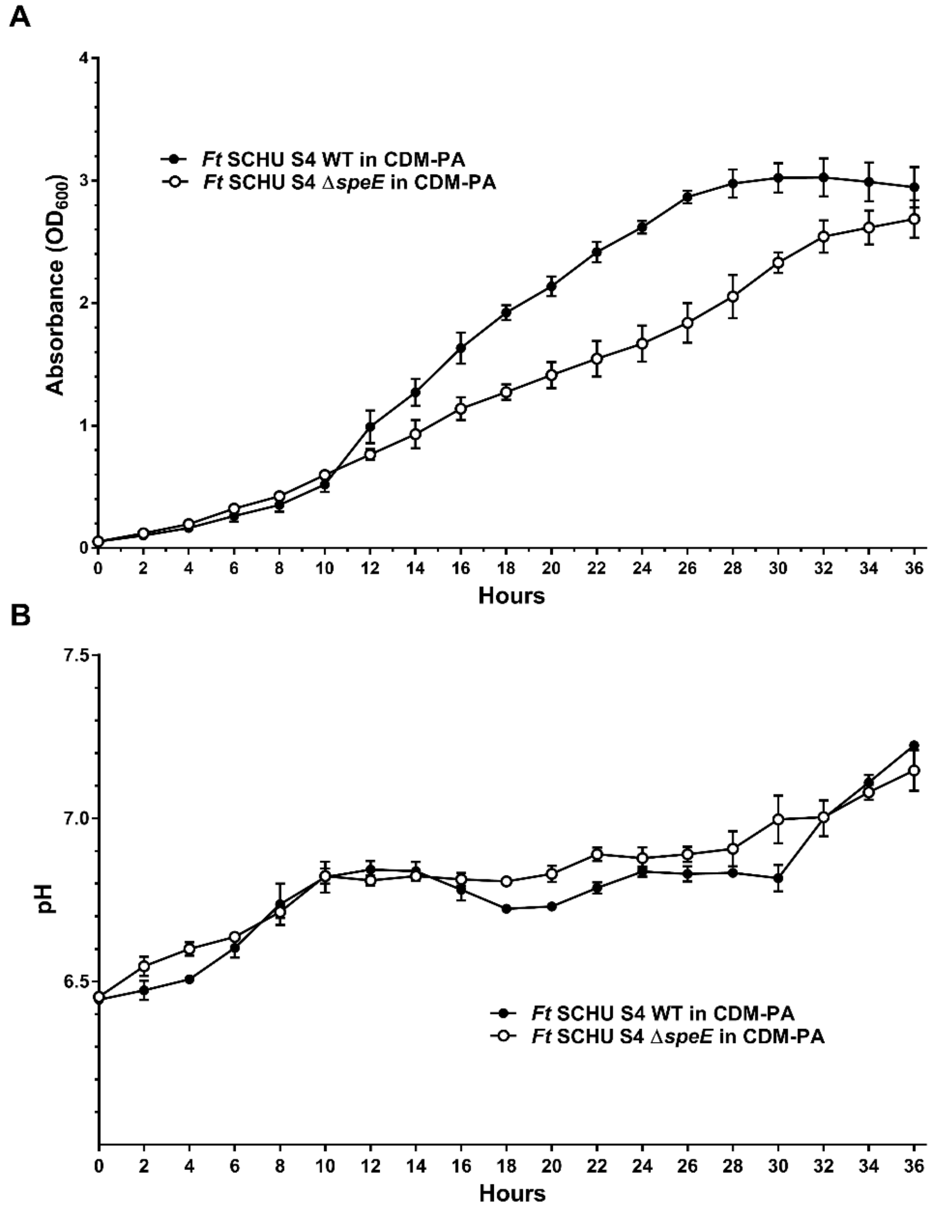

FIGURE S6 Evaluation of culture media pH during growth of *F. tularensis* SCHU S4 and the isogenic Δ*speE* mutant. *F. tularensis* (*Ft*) strains were grown with aeration at 37°C in flasks containing chemically defined medium without any polyamines (CDM-PA), and **(A)** the cell density based on absorbance readings and **(B)** the pH of the culture medium was determined every two hours using a micro pH electrode. Mean ± SEM are shown for three biological replicates in two independent experiments.

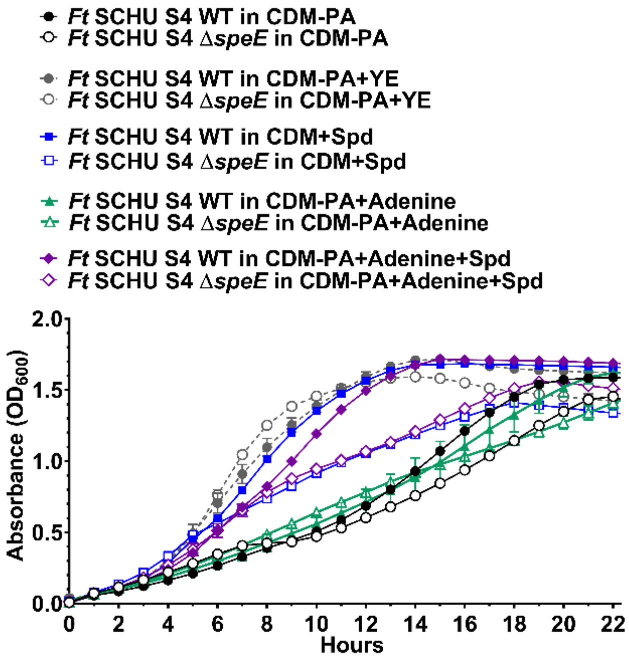

FIGURE S7 Growth of *F. tularensis* SCHU S4 and isogenic Δ*speE* mutant in chemically defined medium without any polyamines and with or without yeast extract, adenine, and/or spermidine supplementation. Shown is the growth of SCHU S4 wildtype (WT, filled symbols) and the SCHU S4 Δ*speE* mutant (unfilled symbols) in chemically defined medium without any polyamines (CDM-PA, black circles), with yeast extract (CDM-PA+YE, gray circles), with 0.1 mM spermidine (CDM+Spd, blue squares), with 0.1 mM adenine (CDM+Adenine, green triangles), and with both 0.1 mM adenine and 0.1 mM spermidine (CDM+Adenine+Spd, purple diamonds). Data represents the mean ± SEM error bars of three biological replicates in each of three independent experiments.
